# Continuous vs Intermittent Postoperative Vital Sign Monitoring: A Cluster Randomized Crossover Trial

**DOI:** 10.1001/jamanetworkopen.2026.3290

**Published:** 2026-03-26

**Authors:** Ashish K. Khanna, Nathaniel Sean O’Connell, Amit K. Saha, Megan Henley Hicks, Robert S. Weller, Lynnette Harris, Bruce D. Cusson, Ann Faris, Carolyn S. Huffman, Scott Segal, Brian J. Wells, Eric S. Kirkendall, Daniel I. Sessler

**Affiliations:** 1 Division of Critical Care Medicine, Department of Anesthesiology, Wake Forest University School of Medicine, Atrium Health Wake Forest Baptist Medical Center, Winston-Salem, North Carolina; 2 Perioperative Outcomes and Informatics Collaborative, Winston-Salem, North Carolina; 3 Outcomes Research Consortium, Houston, Texas; 4 Division of Public Health Sciences, Department of Biostatistics and Data Science, Wake Forest University School of Medicine, Winston-Salem, North Carolina; 5 Division of Cardiac Anesthesiology, Department of Anesthesiology, Wake Forest University School of Medicine, Atrium Health Wake Forest Baptist Medical Center, Winston-Salem, North Carolina; 6 Division of Regional Anesthesia and Pain Medicine, Department of Anesthesiology, Wake Forest University School of Medicine, Atrium Health Wake Forest Baptist Medical Center, Winston-Salem, North Carolina; 7 Department of Implementation Science, Wake Forest University School of Medicine, Winston-Salem, North Carolina; 8 Department of Pediatrics, Wake Forest University School of Medicine, Atrium Health Wake Forest Baptist Medical Center, Winston-Salem, North Carolina; 9 Center for Healthcare Innovation, Wake Forest University School of Medicine, Atrium Health Wake Forest Baptist Medical Center, Winston-Salem, North Carolina; 10 Center for Outcomes Research and Department of Anesthesiology, The University of Texas Health Science Center at Houston (UTHealth Houston); 11 Department of Nursing, Atrium Health Wake Forest Baptist Medical Center, Winston-Salem, North Carolina

## Abstract

**Importance:**

Continuous postoperative monitoring on general nursing units detects vital sign abnormalities that are missed with conventional intermittent monitoring. Early recognition may prompt interventions to limit potentially harmful blood pressure, heart rate, and oxygen saturation perturbations.

**Objective:**

To test the hypothesis that continuous unblinded monitoring of vital signs would decrease blood pressure, heart rate, and oxygen saturation abnormalities compared with intermittent monitoring in patients recovering from noncardiac surgery.

**Design, Setting, and Participants:**

This multiple crossover randomized cluster trial in 2 postoperative hospital wards included patients recovering from noncardiac surgery and was conducted from October 7, 2020, to October 7, 2021. Data were analyzed from January to July in 2025.

**Intervention:**

One participating ward was randomized to an initial month of either unblinded or clinician-blinded continuous vital sign monitoring and thereafter alternated at 4-week intervals for 1 year; the other ward always used the alternate monitoring approach. All patients had intermittent vital signs always available, whereas continuous vital signs were only available to clinicians during unblinded weeks. When continuous vital signs were unblinded, alerts at a mean arterial pressure less than 65 mm Hg, an oxygen saturation less than 90%, and a heart rate more than 110 per minute were transmitted to treating teams.

**Main Outcomes and Measures:**

The primary outcomes were durations of hypotension, hypoxemia, and tachycardia exceeding alert thresholds during the initial 48 hours of ward admission or until the third postoperative morning. Secondary outcomes were interventions categorized, with increasing severity, as none, independent nursing intervention, physician notification, or activation of the hospital emergency or rapid response systems.

**Results:**

Among 798 total patients (median [IQR] age, 70.7 [62.5-78.7] years; 440 females [55%]), who included 404 assigned to continuous monitoring and 394 to intermittent monitoring, 561 (70%) with the American Society of Anesthesiologists’ physical status III were included in the final analysis. The duration of oxygen saturation less than 90% was reduced by approximately 30 minutes over 48 hours of monitoring time, in continuously monitored patients (unblinded continuous monitoring group median [IQR], 70.8 [23.1-154.2] minutes vs blinded intermittent group median [IQR], 103.5 [29.5-249.7] minutes; geometric means ratio, 0.86 [95% CI, 0.78-0.95]; *P* = .002). Hypotension and tachycardia durations were not significantly reduced. The number of interventions was similar, with approximately half in each group receiving new oxygen therapy (unblinded continuous monitoring: 224 [55%] and blinded intermittent monitoring: 214 [54%]).

**Conclusions and Relevance:**

In this cluster randomized crossover trial of continuous compared with intermittent vital sign monitoring, continuous monitoring reduced the duration of desaturation in patients recovering from noncardiac surgery on general hospital wards. Reducing the duration of vital sign abnormalities may decrease the risk of more serious complications, and robust patient-centric outcomes trials seem warranted.

**Trial Registration:**

ClinicalTrials.gov Identifier: NCT04574908

## Key Points

**Question:**

Does continuous vital sign monitoring reduce abnormalities in blood pressure, oxygenation, and heart rate during the initial 48 hours after noncardiac surgery?

**Findings:**

In this cluster randomized trial, using 4-week ward clusters among 798 postoperative patients, the duration of oxygen saturation less than 90% was reduced by approximately 30 minutes in continuously monitored patients, whereas hypotension and tachycardia durations did not decrease significantly.

**Meaning:**

The findings of this study suggest that continuous postoperative ward monitoring reduces hypoxemia and warrants robust patient-centric outcomes trials.

## Teaser

This cluster trial assesses whether continuous monitoring of vital signs decreases duration of blood pressure, heart rate, and oxygen saturation abnormalities compared with intermittent monitoring in patients recovering from noncardiac surgery.

## Introduction

Postoperative hypotension is common, serious, and often prolonged.^1,2^ For example, 10% of patients with clinician-blinded continuous vital sign monitoring have at least 15 continuous minutes with mean arterial pressures (MAPs) less than 65 mm Hg over the initial 48 postoperative hours. Importantly, nursing vital sign assessments at 4-hour intervals miss such episodes in half of the patients.^3^ In the POISE-2 (Perioperative Ischemic Evaluation-2) trial, for example, more than 40% of participating patients had postoperative systolic pressures less than 90 mm Hg, as detected by routine intermittent nursing evaluations, and hypotension was associated with a 3-fold increase in mortality.^4^

Respiratory complications are also preventable causes of postoperative morbidity and mortality. Hypoxemia and ventilatory impairment are common in postoperative patients and are underestimated with intermittent monitoring.^5,6^ For example, with clinician-blinded continuous postoperative monitoring, 21% of inpatients recovering from noncardiac surgery had at least 10 minutes with oxygen saturation (SpO_2_) less than 90% per hour.^5^ More than 90% of episodes, during which saturation was less than 90% for a continuous hour, were missed by the nurses making routine measurements at 4-hour intervals.^5^ Analysis of the malpractice closed-claims database indicates that half of the reported postoperative respiratory events were lethal, and a quarter caused serious neurologic injury. Almost all of these events were deemed preventable with a better monitoring and response system.^7,8^ It is also likely that both desaturation and hypotension contribute to myocardial injury, which largely results from a supply-demand mismatch.^9^

There is now considerable evidence that vital sign abnormalities occur hours before acute cardiac or respiratory arrests.^10,11,12^ Early detection and appropriate treatment may prevent future catastrophic events. However, clinical deterioration is often missed when vital signs are evaluated only intermittently. Continuous postoperative monitoring and associated nursing alerts detect cardiorespiratory compromise in real time and may prompt clinical interventions that possibly prevent progression to critical events. Nonetheless, continuous ward monitoring is challenged by alarm fatigue and paucity of trials to support its benefit. We therefore tested the primary hypothesis that continuous monitoring of vital signs would decrease vital sign abnormalities compared with intermittent monitoring in patients recovering from noncardiac surgery. Secondarily, we tested the hypothesis that increasing bedside clinical nursing interventions (ranging from no response to activation of rapid response teams) would be more common in patients randomized to unblinded continuous monitoring and associated alerts.

## Methods

This cluster trial was approved by the Wake Forest institutional review board. Individual informed consent was waived because no patients were denied routine monitoring or care, and the intervention (continuous vital sign monitoring) was deemed low-risk and potentially beneficial. The trial was conducted with active collaboration from relevant nursing teams, surgeons, resident physicians, and emergency medical response team members. Before starting, relevant clinicians thus had a good understanding of the trial protocol (Supplement 1) and their roles. All results reported adhered to the Consolidated Standards of Reporting Trials (CONSORT) reporting guideline.

### Protocol and Measurements

We enrolled adults aged 18 years or older who were admitted to the Atrium Health Wake Forest Baptist Medical Center between October 7, 2020, and October 7, 2021, and assigned to 2 designated postoperative wards. Perioperative management was entirely per clinical routine and included general and regional anesthesia, along with various types of postoperative analgesia. Additionally, all participating patients were continuously monitored throughout hospitalization with ViSi Mobile (Sotera Wireless), a portable wrist-mounted system that provides continuous ambulatory vital sign monitoring and has been cleared by the US Food and Drug Administration.

The portable vital sign monitoring device measured and recorded SpO_2_, heart rate, respiratory rate, 5-lead electrocardiogram, and noninvasive blood pressure (BP) estimated from pulse transit time. The monitoring device’s estimated maximal mean (SD) error was 5 (8) mm Hg. However, the monitoring device’s BP assessments had not been externally validated. The device’s monitors were calibrated daily and connected to the hospital’s wireless network.

We compared 2 monitoring methods. The first was blinded continuous vital sign monitoring combined with vital signs obtained manually at 4-hour intervals. Nursing station monitoring device screens were turned off, patient wrist monitors were covered, and alarms were disabled. Effectively, this approach corresponded to routine management except that fail-safe alerts were generated for extreme vital signs, namely a heart rate more than 150 per minute, a systolic BP less than 70 mm Hg, and an SpO_2_ less than 80%.

Our second method was manual vital signs at 4-hour intervals combined with unblinded continuous vital sign monitoring with values displayed on patient monitors and at the corresponding nursing station. Our prespecified alert thresholds were a MAP less than 65 mm Hg, a heart rate more than 110 per minute, and an SpO_2_ less than 90%. Vital signs exceeding thresholds generated alerts that were distributed to a central station and to the nurses’ hospital-supplied phones. Alarms that were not addressed by the primary nurses escalated to other floor nurses and thereafter to the unit manager per hospital protocol.

Two hospital wards (approximately 25 beds each) were designated as trial clusters. The initial monitoring strategy was randomly allocated for 1 ward with the other participating ward being assigned to the alternate strategy. Thereafter, monitoring approaches alternated in each ward at 4-week intervals for 1 year (eTables 1 and 2 in Supplement 2). Clinicians were asked to order serial high-sensitivity troponin I on the first 3 postoperative days while patients remained hospitalized, with values more than 40 pg/mL considered evidence of myocardial injury after noncardiac surgery (MINS).

Our primary outcome was the duration of hypotension (MAP <65 mm Hg), oxygen desaturation (SpO_2_ <90%), and tachycardia (heart rate >110 per minute). Secondary outcomes were clinical nursing interventions graded on a scale of 1 to 4 with increasing severity and categorized as: (1) none, (2) independent nursing intervention, (3) physician notification, or (4) activation of the hospital emergency or rapid response system.

MINS was an exploratory outcome and defined by any high-sensitivity troponin I concentration more than 40 pg/mL during the initial 3 postoperative days. We also considered 2 exploratory post hoc composites. Composite A was a MAP less than 65 mm Hg, a heart rate more than 110 per minute, or an SpO_2_ less than 90%. Composite B included any of the components in composite A along with either hypertension (MAP >130 mm Hg) or bradycardia (heart rate <50 per minute). On an exploratory basis, we report individual composite components along with an analysis based on each of the thresholds of a MAP less than 70 mm Hg, a MAP less than 75 mm Hg, and a MAP less than 80 mm Hg.

Race and ethnicity were self-reported and extracted from electronic health records for use in this study because the participants were not prospectively asked for this information. Race and ethnicity categories included American Indian or Alaska Native, Asian, Black or African American, White, and other (Latin American or Hispanic and Native Hawaiian or Other Pacific Islander).

### Statistical Analysis

Data were analyzed from January to July in 2025. We enrolled adults recovering from noncardiac surgery who were admitted to the designated wards and expected to stay at least 48 hours. However, our planned analysis was restricted to a population with an increased risk of adverse cardiovascular outcomes. Therefore, we only included adults aged 65 years or older or aged 45 years or older who had at least 1 cardiovascular risk factor (eg, hypertension, diabetes) who had a general or regional anesthetic in the final analysis.

Continuous vital signs were recorded at 15-second intervals, and the fraction of measurements that exceeded designated thresholds over the initial 48 hours of continuous monitoring were calculated and are presented as medians (IQRs). In each model, we adjusted for patient-level variables including age, sex, race and ethnicity, diabetes, do-not-resuscitate status, hypertension, American Society of Anesthesiologists’ physical status, body mass index, in-hospital mortality, and intensive care unit (ICU) transfer. Results are presented as a ratio of geometric means (GMR) estimates from these models and 95% CIs, along with median durations of each outcome and intergroup differences. Additionally, we evaluated the binary outcome of MINS with a generalized linear model, assuming a Poisson distribution and log-link function with a robust variance estimator to yield a risk ratio outcome.

To avoid the composite outcome estimates being driven by high-frequency components (such as oxygen therapy) compared with much rarer components (such as in-hospital mortality) for the secondary outcomes of clinical interventions, we used a multivariable generalized linear model with logit link and an unstructured correlation for components of the composite outcome to estimate the average relative effect, adjusting for potential confounders (generalized estimating equation model).^13^ We compared this with a general regression model with equal weightage for all of the components of clinical interventions as a composite. All analyses were performed with generalized linear mixed-effects models fit using the package GLMMadaptive in R, version 4.10 (R Project for Statistical Computing).^14,15^ A 2-sided *P* < .05 indicated statistical significance.

As is standard in the clustered study design literature, we performed a power calculation that assumed individual-level randomization and then adjusted for cluster-level correlation because study participants cannot be viewed as statistically independent within a cluster and were not individually randomized. Accounting for potential intracluster correlation within each ward of 0.12 and a reduction in abnormal vital signs of 0.42 (informed from preliminary data), we planned to enroll 500 patients per group.

## Results

**Figure 1.**
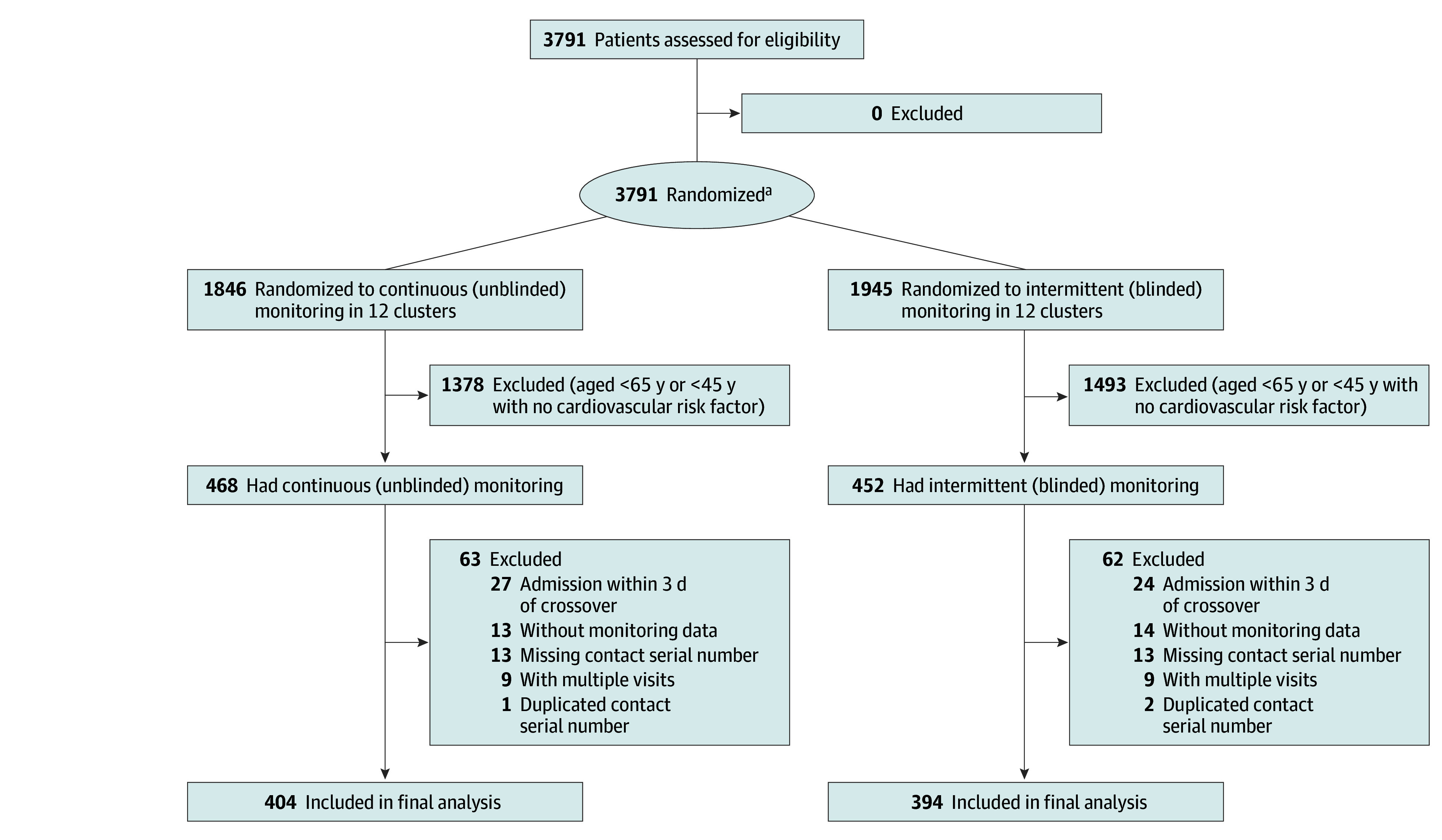
CONSORT Flow Diagram for the Trial Randomized patients recovering from noncardiac surgery were expected to stay at least 48 hours in the hospital. ^a^Categories for exclusion were not mutually exclusive.

Over the 1-year trial period, we pragmatically included 3791 adults recovering from noncardiac surgery who had planned hospital stays of 48 hours or more. Among them, 1945 had intermittent (blinded continuous) monitoring and 1846 continuous (unblinded) monitoring (Figure 1). We planned alternating interventions with 4-week blocks over the course of a calendar year, but owing to administrative and technical issues, there were 12 exposure blocks that lasted between 26 and 37 days over 365 days (eTables 1 and 2 in Supplement 2).

All randomized patients who had at least some continuous monitoring were enrolled. However, per protocol, analysis was restricted to patients with high risk, specifically those aged 65 years or older or 45 years or older with at least 1 cardiovascular risk factor (eg, hypertension, diabetes). There were thus 468 patients who qualified for analysis with unblinded continuous monitoring and 452 with intermittent (blinded continuous) monitoring. After application of other exclusion criteria, a total of 798 patients (median [IQR] age, 70.7 [62.5-78.7] years; 440 females [55%] and 358 males [45%]) were included in the final analyses: 404 with continuous monitoring and 394 with blinded monitoring (Figure 1). Among the total patients, 561 (70%) were included with the American Society of Anesthesiologists’ physical status III, a grading system ranging from I to VI, with higher grades indicating more severe disease.

Detailed demographic and morphometric characteristics of the trial cohort are shown in Table 1. Baseline variables were comparable across groups. Of the total patients, 4 (0.5%) were American Indian or Alaska Native, 4 (0.5%) were Asian, 128 (16.0%) were Black or African American, 641 (80.3%) were White, and 21 (2.6%) were of other race and ethnicity. The median (IQR) duration of monitoring time was 80.83 (49.38-123.93) hours in both of the intervention groups. The intracluster correlation among nursing wards was 0 for each outcome of interest. We thus had considerably more power than anticipated, despite enrolling approximately 20% fewer qualifying patients than planned.

**Table 1.** Baseline and Demographic Characteristics of the Trial Population

Characteristic	Patients, No. (%)	
	Continuous monitoring (unblinded) (n = 404)	Intermittent monitoring (blinded) (n = 394)
Age, median (IQR), y	70.5 (62.4-78.0)	71.2 (63.3-80.1)
Sex		
Female	223 (55.2)	217 (55.1)
Male	181 (44.8)	177 (44.9)
Race and ethnicity		
American Indian or Alaska Native	3 (0.7)	1 (0.3)
Asian	1 (0.2)	3 (0.8)
Black or African American	65 (16.1)	63 (16.0)
White	324 (80.2)	317 (80.5)
Other^a^	11 (2.7)	10 (2.5)
American Society of Anesthesiologists PS		
I	0	1 (0.3)
II	33 (8.2)	37 (9.5)
III	291 (72.6)	270 (69.2)
IV	72 (18.0)	75 (19.2)
V	5 (1.2)	7 (1.8)
Diabetes		
Yes	137 (33.9)	124 (31.5)
No	267 (66.1)	270 (68.5)
Do-not-resuscitate status		
Yes	32 (7.9)	41 (10.4)
No	372 (92.1)	353 (89.6)
Hypertension		
Yes	317 (78.5)	307 (77.9)
No	87 (21.5)	87 (22.1)
BMI, median (IQR)	29.4 (24.9-34.7)	29.3 (25.0-34.9)
Hospital length of stay, median (IQR), d	4.3 (2.5-6.9)	4.7 (2.7-7.6)
Monitoring time followed, median (IQR), h	78.4 (50.2-121.0)	82.3 (48.2-124.2)

Abbreviations: BMI, body mass index (calculated as weight in kilograms divided by height in meters squared); PS, physical status.

^a^ Includes Latin American or Hispanic or Native Hawaiian and Other Pacific Islander.

**Figure 2.**
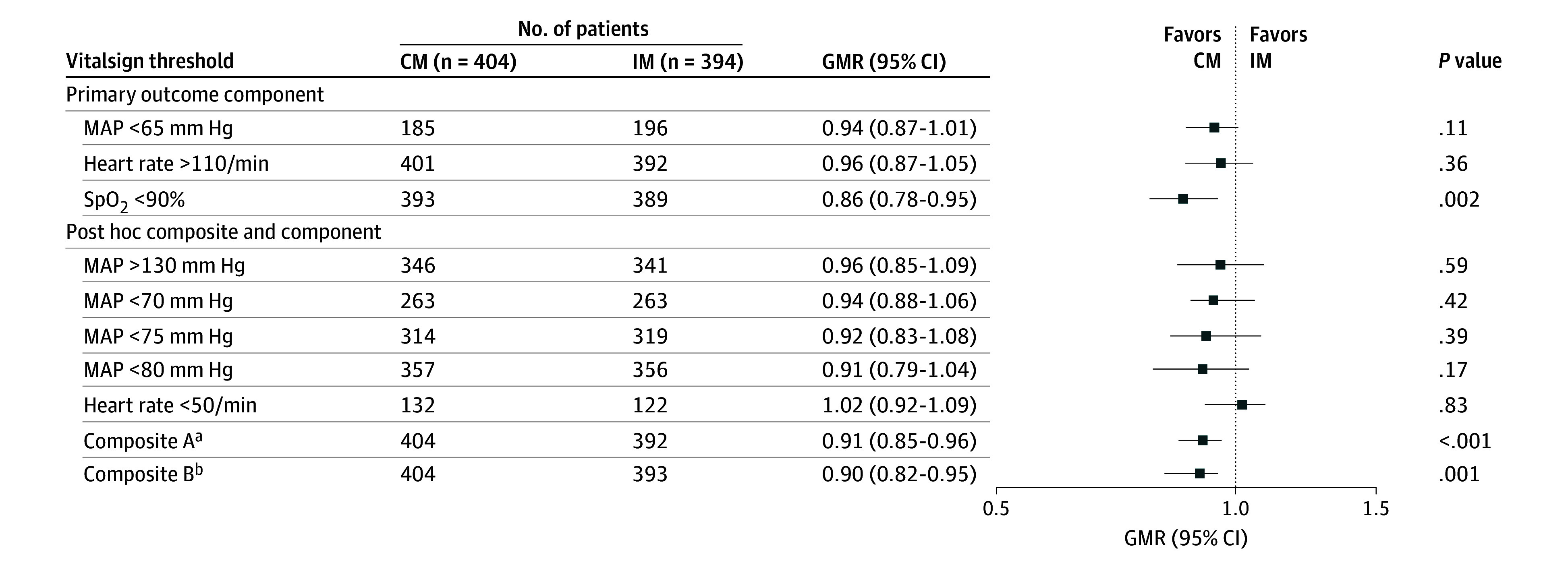
Forest Plot of the Geometric Means Ratio (GMR) of Exceeding Critical Vital Sign Thresholds in Continuous Monitoring (CM) vs Intermittent Monitoring (IM) Generalized linear model results were adjusted for age, sex, race and ethnicity, diabetes, hypertension, do-not-resuscitate order status, in-hospital mortality, and intensive care unit transfer. MAP indicates mean arterial pressure; SpO_2_, oxygen saturation. ^a^MAP less than 65 mm Hg, heart rate more than 110 per minute, or SpO_2_ less than 90%. ^b^Any of the components in composite A and MAP more than 130 mm Hg or heart rate less than 50 per minute.

The time spent beyond the thresholds for each outcome, the intergroup differences, GMRs, 95% CIs, and *P* values from each of the adjusted models are shown in Figure 2 and Table 2. We demonstrate the percentage of patients (y-axis) who experienced a percentage of time (x-axis) beyond each relevant critical threshold by monitoring group in eFigures 1 to 8 in Supplement 2. For example, for SpO_2_, about 30% of patients who were intermittently monitored (blinded) spent at least 10% of time with an SpO_2_ less than 90%, while only about 10% of patients who were continuously monitored (unblinded) had an SpO_2_ less than this threshold for at least 10% of monitored time (eFigure 1 in Supplement 2).

**Table 2.** Durations of Outcomes and Risk of Exceeding Critical Vital Sign Thresholds in the Continuous Monitoring Group vs Intermittent Routine Monitoring^a^

Outcome	Duration min per 48 h, median (IQR)		Intergroup difference, median (IQR)	GMR (95% CI)^b^	*P* value^c^
	Continuous monitoring (unblinded) (n = 404)	Intermittent monitoring (blinded) (n = 394)			
MAP, mm Hg					
<65	0 (0 to 2.9)	0 (0 to 4.4)	0 (0 to 1.5)	0.94 (0.87 to 1.01)	.11
<70	2.1 (0 to 21.1)	2.3 (0 to 27.9)	0.2 (0 to 6.8)	0.94 (0.88 to 1.06)	.42
<75	14.5 (0.8 to 87.5)	18.8 (0.8 to 110.6)	4.3 (0 to 23.1)	0.92 (0.83 to 1.08)	.39
<80	69.3 (6.8 to 293.9)	103.6 (11.4 to 277.0)	34.3 (4.6 to −16.9)	0.91 (0.79 to 1.04)	.17
>130	48.2 (3.8 to 133.2)	48.7 (3.8 to 165.6)	0.5 (0 to 32.4)	0.96 (0.85 to 1.09)	.59
Heart rate, per min					
>110	66.0 (19.7 to 187.1)	72.6 (23.7 to 194.9)	6.6 (4.0 to 7.8)	0.96 (0.87 to 1.05)	.36
<50	0 (0 to 0.4)	0 (0 to 0.2)	0 (0 to −0.2)	1.02 (0.92 to 1.09)	.83
SpO_2_ <90%	70.8 (23.1 to 154.2)	103.5 (29.5 to 249.7)	32.7 (6.4 to 95.5)	0.86 (0.78 to 0.95)	.002
Composite A^d^	232.2 (115.7 to 446.3)	311.4 (149.1 to 565.6)	79.2 (33.4 to 119.3)	0.91 (0.85 to 0.96)	.001
Composite B^e^	352.8 (190.9 to 591.6)	428.4 (252.4 to 732.2)	176.0 (−479.8 to 379.4)	0.90 (0.82 to 0.95)	<.001

Abbreviations: GMR, geometric means ratio; MAP, mean arterial pressure; SpO_2_, oxygen saturation.

^a^ Multiple regression model adjusted for age, sex, race and ethnicity, diabetes, hypertension, do-not-resuscitate order status, American Society of Anesthesiologists physical status, body mass index, in-hospital mortality, and intensive care unit transfer.

^b^ GMR less than 1 favors continuous (unblinded) monitoring.

^c^ *P* value for GMR.

^d^ MAP less than 65 mm Hg, SpO_2_ less than 90%, or heart rate more than 110 per minute.

^e^ Composite A and heart rate less than 50 per minute or MAP more than 130 mm Hg.

During the initial 48 hours of monitoring, the duration of SpO_2_ less than 90% was reduced by approximately 30 minutes in continuously monitored patients (unblinded continuous monitoring group median [IQR], 70.8 [23.1-154.2] minutes vs blinded intermittent group median [IQR], 103.5 [29.5-249.7] minutes; intergroup difference median [IQR], 32.7 [6.4-95.5] minutes; GMR, 0.86 [95% CI, 0.78-0.95]; *P* = .002). The amounts of tachycardia and hypotension were not significantly reduced (Table 2).

Composite outcome A combined any of the components of the primary outcome (SpO_2_ <90%, heart rate >110 per minute, or MAP <65 mm Hg) and composite outcome B additionally included a heart rate less than 50 per minute or a MAP more than 130 mm Hg. Both were significantly improved by continuous monitoring with GMRs of 0.91 (95% CI, 0.85-0.96) and 0.90 (95% CI, 0.82-0.95). All components of both composites had GMRs less than 1, but none was individually statistically significant.

Our secondary outcome could not be accessed as originally specified in the trial protocol because interventions performed at the bedside by nursing staff were inadequately documented. However, oxygen therapy, naloxone use, rapid response calls, ICU transfers, in-hospital mortality, and a composite with any of these were reported and did not differ significantly (Table 3 and eFigure 9 in Supplement 2). Specifically, 320 patients (79.2%) in the continuous monitoring group and 308 (78.2%) in the intermittent monitoring group had at least 1 intervention (adjusted relative risk [ARR], 0.92 [95% CI, 0.78-1.08]; *P* = .21). The most common intervention was initiation of new oxygen therapy for 224 patients (55%) with unblinded and 214 patients (54%) with blinded monitoring (ARR, 1.01 [95% CI, 0.92-1.15]; *P* = .82). An important reason for nonescalation of therapy could be alarm fatigue. Alarm thresholds for a heart rate more than 110 per minute and an SpO_2_ less than 90 were reached most frequently followed by a MAP less than 65 mm Hg (eTable 3 in Supplement 2).

**Table 3.** Clinical Outcomes and Relative Risk in the Continuous and Intermittent Monitoring Groups

Outcome	Patients, No. (%)		ARR (95% CI)^a^	*P* value
	Continuous monitoring (unblinded) (n = 404)	Intermittent monitoring (blinded) (n = 394)		
In-hospital mortality	4 (1)	7 (2)	0.52 (0.36-1.03)	.13
ICU transfer	21 (5)	31 (8)	0.69 (0.41-1.17)	.17
Rapid response call	33 (9)	44 (12)	0.73 (0.49-1.11)	.21
Naloxone use	37 (9)	55 (14)	0.71 (0.48-1.04)	.22
Oxygen therapy	224 (55)	214 (54)	1.01 (0.92-1.15)	.82
General regression model composite^b^	248 (61)	246 (62)	0.96 (0.88-1.12)	.76
GEE model composite	NA	NA	0.83 (0.64-1.06)	.18

Abbreviations: ARR, adjusted relative risk; GEE, generalized estimating equation; ICU, intensive care unit; NA, not applicable.

^a^ Adjusted for body mass index, American Society of Anesthesiologists physical status, age, sex, race and ethnicity, diabetes, do-not-resuscitate order status, and hypertension.

^b^ Equal weightage to all components.

The exploratory outcome of MINS did not differ across the monitoring groups, with the incidence being 60 (14.9%) with unblinded monitoring vs 65 (16.5%) with blinded monitoring (ARR, 1.06 [95% CI, 0.65-1.24]; *P* = .52) (eTable 4 in Supplement 2).

## Discussion

Diseases needing surgical intervention, and as an extension the initial 30 postoperative days, are a leading cause of death.^16^ High mortality may in part result from inadequate vital sign monitoring, which is typically done manually at 4-hour to 6-hour intervals in western hospitals and even less often in many parts of the world. The evolution of vital sign monitoring on hospital wards over the last 50 years has not kept pace with the increasing acuity of the average patient recovering from surgery. It is thus unsurprising that routine intermittent vital sign monitoring often misses many potentially serious abnormalities.^3,6,17^

For example, Sun et al^5^ reported that a fifth of patients who were continuously monitored after noncardiac surgery on general care wards at the Cleveland Clinic Main Campus had 10 minutes or more per hour of an SpO_2_ less than 90%, and nurses missed 90% of the episodes with intermittent monitoring. Similarly, Turan et al^3^ reported that about a fifth of patients on general care wards at the Cleveland Clinic had MAPs less than 65 mm Hg for at least 15 continuous minutes, and intermittent monitoring missed about half of the episodes. The overall duration of vital sign deviations beyond prespecified clinically relevant thresholds was considerably less in patients in our cluster trial than in those reported by Sun et al^5^ and Turan et al^3^ but are consistent with a previous report from the Atrium Health Wake Forest Baptist Medical Center, in which only about 10% of patients spent 15 continuous minutes at MAPs less than 65 mm Hg when monitored continuously.^17^ Varying amounts of vital sign abnormalities presumably reflect population differences. In addition, whereas Sun et al^5^ and Turan et al^3^ were blinded continuous-monitoring studies, the recent report from our institution^17^ was an analysis of nearly 15 000 patients with unblinded monitoring with alarms activated and available for nursing intervention. The fact that Wake Forest uses continuous vitals monitoring routinely may have also influenced the overall duration of vital sign deviations in both of our cohorts.

In our study, continuous vital sign monitoring of adult patients in surgical wards decreased the risk of hypoxemia by 14%. Continuous monitoring was also also estimated to decrease hypotension by approximately 6% and tachycardia 4%, although these differences were not statistically significant. A composite of hypotension, tachycardia, or hypoxemia was reduced by 9% with continuous monitoring, and another composite, which included hypertension or bradycardia, was reduced by 10%.

BP and heart rate abnormalities were similar in patients assigned to blinded and unblinded continuous postoperative vital sign monitoring. In contrast, the duration of desaturation (SpO_2_ <90%) was about 30 minutes less in patients randomized to unblinded continuous ward monitoring. Interestingly, the fraction of patients given supplemental oxygen was similar with each monitoring strategy, but oxygen was presumably given more effectively in patients randomized to unblinded continuous monitoring.

Our results are generally consistent with a Danish trial that randomized 400 patients to continuous vs intermittent monitoring and reported a mean reduction of desaturation (SpO_2_ <88%) of 47 minutes per day in the continuous monitoring arm.^18^ A larger treatment effect in the Danish group may have resulted from their use of artificial intelligence and a graphic user interface on mobile devices that presumably facilitated information processing. We also note that even though our blinded monitors generated fail-safe alerts at a heart rate more than 150 per minute, a systolic BP less than 70 mm Hg, and an SpO_2_ less than 80%, the times spent beyond these thresholds were less than 0.05 minutes across both groups, and this likely did not diminish the apparent differences between the treatment groups. Our results are also generally consistent with a 150-patient trial, in which patients were randomized to blinded or unblinded continuous ward monitoring.^19^ Available literature thus suggests that continuous ward monitoring identifies more vital sign abnormalities and prompts nurses’ interventions that limit the duration and severity of deviations.

Observational analyses suggest that continuous ward monitoring reduces the incidence of postoperative complications including rapid response calls, ICU admissions, and in-hospital mortality.^20,21,22,23,24^ Our results were consistent in that we had an approximately 30% relative reduction in rapid response activations and ICU transfers in patients assigned to continuous monitoring. Curiously, an important intervention for desaturation is oxygen supplementation, which was similar in both arms. It is likely that bedside nursing-driven early microinterventions (such as relieving an obstructed airway by repositioning, stimulation, and encouraging deep breathing) were more common in the unblinded monitoring arm and closed the mechanistic loop between available alarms and improved SpO_2_. Although our pilot trial was underpowered for these exploratory outcomes by at least an order of magnitude, they are highly encouraging and suggest that unblinded continuous ward monitoring might improve substantive outcomes. A larger trial to evaluate the effect of continuous postoperative ward monitoring on such highly clinically meaningful complications seems well worth considering.

MINS is strongly associated with postoperative hypotension, typically silent and only detected by troponin elevations. Thirteen percent of all postoperative deaths are attributed to it.^25,26^ Our theory therefore is that continuous monitoring identifies hypotension and that nurses would intervene to limit the amount and severity of hypotension, thereby reducing myocardial injury. However, hypotension was uncommon in the patients in our study, with fewer than 1% of patients in either group with a MAP less than 65 mm Hg; even using thresholds of less than 70 mm Hg and less than 75 mm Hg, hypotension was short-lived in both groups. The amount of hypotension that we observed was thus unlikely to provoke myocardial injury. For example, Liem et al^26^ reported that a significant association with MINS required a MAP less than 75 mm Hg to be sustained for 4 hours. Given that there was only sparse hypotension, and similar amounts in each group, it is unsurprising that the incidence of myocardial injury was similar in patients assigned to blinded and unblinded continuous monitoring.

### Limitations

Our study has limitations. This trial had limited generalizability, as it was performed at a single center, and clusters were limited to 2 hospital wards only. An advantage of cluster-design trials is that all patients within clusters are automatically and efficiently enrolled. Our trial maintained a pragmatic approach, in which we tested the effect of the type of ward monitoring in a clinical setting, allowing practitioners to intervene based on their best clinical judgement. A consequent disadvantage is that enforcing the protocol across patients and clinicians is often challenging. Adherence matters because even limited nonadherence can make results unreliable and markedly reduce power. For example, troponin monitoring was requested per protocol but ultimately at clinician discretion (eTable 4 in Supplement 2). Given that myocardial injury is a sparse outcome, our trial was seriously underpowered for this exploratory outcome and could be regarded as a pilot trial, which is largely hypothesis-generating for this outcome. We could not prospectively collect practitioner response to alarms data as planned in the secondary outcome because of the lack of accurate and real-time documentation. As a surrogate, we collected clinical interventions, which were only rough estimates of how clinical teams responded to detected vital sign changes. We did not collect data on signal quality or periods of time without monitoring on individual patients, although we did exclude patients without any monitoring data.

It remains unclear whether just 30 minutes of SpO_2_ less than 90% over 48 hours is clinically meaningful. However, nonsignificant improvements in hard outcomes including ICU transfer, rapid response team calls, and naloxone use suggest that extra patient attention consequent to continuous monitoring may produce meaningful clinical improvements. There was no way to avoid an obvious Hawthorne effect in that all personnel on designated wards knew that the trial was ongoing, which could have modified response to alarms in both arms. We also had no process to accurately document nursing responses in the unblinded arm or to evaluate alarm fatigue.

## Conclusions

In this cluster randomized crossover trial of continuous compared with intermittent vital sign monitoring, continuous monitoring reduced the duration of desaturation in patients recovering from noncardiac surgery on general hospital wards. Vital sign abnormalities were less common than previously reported in patients with potentially more severe illness. However, abnormalities remained common. All vital signs improved with unblinded continuous vital sign monitoring, although only desaturation improved significantly. Improvement presumably resulted when alerted nurses intervened, with supplemental oxygen being the most common intervention. It is plausible, and perhaps likely, that reducing the duration of vital sign abnormalities may reduce more serious complications. While our trial lacked power to evaluate complications, our results suggest that robust larger trials evaluating hard outcomes are warranted, and human factors in response to enhanced ward monitoring systems need due consideration.

## Notes

Supplement 1.
Trial Protocol

Supplement 2. **eFigure 1.** Percentage of Patients With Time Beyond an SpO_2_ <90% **eFigure 2.** Percentage of Patients With Time Beyond a MAP <65 mm Hg **eFigure 3.** Percentage of Patients With Time Beyond a Heart Rate >110/Minute **eFigure 4.** Percentage of Patients With Time Beyond a Heart Rate <50/Minute **eFigure 5. **Percentage of Patients With Time Beyond a MAP <70 mm Hg **eFigure 6.** Percentage of Patients With Time Beyond a MAP <75 mm Hg **eFigure 7.** Percentage of Patients With Time Beyond a MAP <80 mm Hg **eFigure 8.** Percentage of Patients With Time Beyond a MAP >130 mm Hg **eFigure 9.** Adjusted Relative Risk Ratios of Intervention in Continuous Monitoring Relative to Intermittent Monitoring **eTable 1.** Schematic for Planned Alternating Interventions Trial **eTable 2.** Actual Days and Patients in Each Cluster of the Alternating Interventions **eTable 3.** Frequency of Unfiltered Alarms During the Trial Period for the Set Thresholds **eTable 4.** Troponin and Myocardial Injury After Noncardiac Surgery (MINS)

Supplement 3.
Data Sharing Statement
