# Supplementary material for: Continuous vs Intermittent Postoperative Vital Sign Monitoring: A Cluster Randomized Crossover Trial
Source: JAMA Netw Open. 2026 Mar 26;9(3):e263290. doi: 10.1001/jamanetworkopen.2026.3290 (PMC13022735; doi:10.1001/jamanetworkopen.2026.3290)
Supplement: Supplement 1. — Trial Protocol [file jamanetwopen-e263290-s001.pdf]

Study Title: A Pilot Trial of Continuous Portable Postoperative Hemodynamic And Saturation Monitoring On Hospital Wards

Principal Investigator, Co-investigator(s): Ashish K. Khanna, MD., FCCP.,FCCM, Associate Professor, Section Head for Research, Department of Anesthesiology, Section on Critical Care Medicine, Wake Forest Baptist Medical Center.

Sponsor or funding source: CTSI KL2 Award

Background, Rationale and Context

Postoperative death within 30 days of surgery is the third most common cause of mortality in this country.(1) The general care floor or hospital ward is traditionally perceived as a place for recovery of postoperative patients who are clinically stable and will transition quickly to leave the hospital. However, about half of all adverse events in hospitalized patients occur on the hospital ward (2-4). Cardiac and respiratory triggers seem to be the most common starting points for these events.(5) A majority of these acute cardiorespiratory events do not occur suddenly. Up to 60% of patients have at least one or more abnormal vital signs starting about 4 hours before an eventual cardiac or respiratory arrest.(4) Early detection of this change in patient physiology therefore becomes critical in effective upstream preventative and therapeutic measures which can translate into improved downstream clinical outcomes. Whereas most patients are still being monitored on an intermittent 'spot check' basis on hospital wards, the evolving role of continuous portable monitoring systems is of obvious benefit in these clinical settings.
At Wake Forest Baptist Medical Center, continuous monitoring of vital signs is as yet not a gold standard of care and is limited to certain units. Most patient assessments are still performed on an intermittent basis with every 4 hourly vital signs checks. As an institution we are leading the way in adoption of portable monitoring technology and have continuous portable monitoring (ViSi device, Sotera Wireless, San Diego, CA) that has been installed in a phased manner for the last 4 years. However, the use of this technology has been incomplete and inconsistent, since there is much question to whether it leads to improved patient outcomes. There is, therefore, availability and familiarity with technology, and also perfect equipoise to conduct the proposed trial. This pilot data will add much needed scientific rigor that is essential for changing and implementing practice guidelines locally and nationally, to help adopt or refute appropriate continuous patient monitoring. This will also form the basis for a much larger multi-center interventional trial of continuous monitoring, which would be the next step to add much needed evidence in this area of work.

Preliminary Studies A recent analysis, led by Dr. Daniel I. Sessler (co-mentor) showed that ward hypoxemia is common, prolonged and profound in both severity and duration. 21% of postoperative non-cardiac inpatients were found to have ≥10 minutes $SpO_2 < 90\%$ *per hour* in patients with clinician-blinded monitoring[11]. More than 90% of desaturation episodes (<90% for a continuous hour) were missed using routine measurements at 4-hour intervals.[11]Patients at highest risk for hypoxemia after surgery, may be those with obstructive sleep apnea and those on narcotic medication for analgesia or a combination of these. The STOP-BANG scoring system is a validated and easy to use mechanism for screening for obstructive sleep apnea (OSA).[12] A higher STOP-BANG score predicts for worsening OSA status and patients with OSA desaturate episodically as a part of the defining criteria of the problem. We monitored patients on the regular ward (with continuous blinded pulse oximetry) for at least 48 postoperative hours, but rather surprisingly found no association between an increasing STOP-BANG

score and postoperative hypoxemia.[13] There was also no relationship between the type of narcotic used in a patient controlled analgesia system (IV hydromorphone and morphine compared with IV fentanyl) and postoperative hypoxemia.[14]These results highlighted the fact that respiratory depression in the postoperative period remains difficult to predict with commonly available tools.[6,15] Several other key preliminary studies have already been published in the Lancet,[9]NEJM,[10] JAMA,[16] and Anesthesiology.[8,17,18] These studies show that hypotension on surgical wards is common and prolonged; that both intraoperative and ward hypotension are strongly associated with myocardial injury and death; that postoperative myocardial infarctions are far more common that generally appreciated and have a 10% 30-day mortality. Dr. Khanna was previously involved in a trial of portable continuous monitoring that showed at least 10% of all hospital ward patients had 15 continuous minutes or more of mean arterial pressures less than 65mmHg over a 48-hour monitoring period. Importantly, nurse initiated spot checks missed detection of hypotension in 50% of these patients. [19] A recent analysis of the PeriOperative ISchemic Evaluation-2 Trial (POISE-2) data showed that exposure to systolic blood pressure less than 90mmHg (as detected using routine nursing evaluation), occurred in 42% of postoperative ward patients and was associated with a nearly 3-fold increase in the odds of mortality.[18] Intraoperative tachycardia is also common to the tune of 71% for a HR>90/min.[20] Dr. Khanna recently finished PRediction of Opioid-induced respiratory Depression In patients monitored by capnoGraphY (PRODIGY), a prospective, observational study of continuous bedside capnography and oximetry conducted in the United States, Europe, and Asia.[21] Hospitalized in patients receiving opioids were included. Monitor alarms and data were blinded. Respiratory compromise episodes were defined by respiratory rate ≤8 bpm for ≥3 minutes; oxygen saturation ≤85% for ≥3 minutes; end-tidal carbon dioxide ≤15 or ≥60 mmHg for ≥3 minutes; apnea episode lasting >30 seconds; or any respiratory Opioid-Related Adverse Events (rORADE). One or more respiratory compromise episodes were detected in 615 (46%) of 1,336 patients over 24 hr (manuscript accepted for publication in Anesthesia & Analgesia). Separate work has shown that half of all closed claims for postoperative respiratory events were lethal and a quarter caused serious neurologic injury. Almost all these events were deemed preventable with a better monitoring and response system.[6,22]
It is thus apparent that postoperative cardio-respiratory events are common and largely unpredictable. It seems highly likely that only continuous hemodynamic/saturation/ventilation monitoring will detect and prevent cardiorespiratory compromise.[6,23-25]

Significance for Patient Safety Currently, there are no standards and limited scientific evidence for patient monitoring on surgical floors. [26] Nationally, standard of care for patient monitoring on hospital wards is intermittent vital signs checks that happen anywhere from once every 4-6 hours. Hospital inpatients represent a large constituency in the health care system—the National Center for Health Statistics estimated a total of 34.7 million discharges in 2005. Accordingly, much effort has historically been expended to keep these patients safe during their stay. In its 2001 report the Institute of Medicine identified failure to rescue—as hospital deaths following adverse occurrences such as post-surgical complications—as a primary patient safety target. The Anesthesia Patient Safety Foundation (APSF) has recently focused significant attention at education and awareness to patient deterioration in the peri surgical period and themed their annual meeting in 2019 as "Perioperative Deterioration: Early Recognition, Rapid Intervention, and the End of Failure to Rescue"

Significance for Bioinformatics, Implementation Science & Innovation for the Future Continuous postoperative monitoring and associated nursing alerts may help detect cardiorespiratory compromise earlier, and in so doing, result in more rapid initiation of correct treatment responses and hospital rapid response systems. These will also generate many thousands of data points which will be connected to patient level data in the electronic medical record. Pilot data from this trial will thus be a rich pool of bioinformatics variables that would be used in the future to develop analytics and machine learning based algorithms to predict who would be likely

to experience a cardiac or respiratory decompensation on the floor. This pilot data would also be critically important to the growth of implementation science and the utilization/improvement of portable continuous monitoring on inpatient units both within and outside this hospital network. The technology used in this trial is futuristic and the trial design is innovative (see under methods) hence the results of this trial will determine the need for future innovations in design & technology that would track our hospitalized patients and their continuum of vital signs during their entire hospital stay and possibly even after their discharge home.

Significance for Wake Forest Baptist Health At Wake Forest Baptist Medical Center, continuous monitoring of vital signs is as yet not a gold standard of care and is limited to certain units. Most patient assessments are still performed on an intermittent basis. As an institution we are leading the way in adoption of portable monitoring technology (ViSi device, Sotera Wireless, San Diego, CA) that has been installed in a phased manner for the last 4 years. However, the use of this technology has been incomplete and inconsistent, since there is much question to whether it leads to improved patient outcomes. There is, therefore, availability and familiarity with technology, and also perfect equipoise to conduct the proposed trial. This pilot data will add much needed scientific rigor that is essential for changing and implementing practice guidelines locally and nationally, to help adopt or refute appropriate continuous patient monitoring.

Objectives

Our primary objective is to show that continuous portable hemodynamic and respiratory monitoring on hospital wards allows clinicians to intervene early and effectively, thus reducing patient exposure to hypotension, tachycardia, and hypoxemia. Continuous monitoring systems on hospital wards should lead to increased nursing interventions, however the danger is false alarms and resultant alarm fatigue will negate any proposed benefit on patient safety outcomes. Therefore, we will access the highest level of nursing response (graded on an ordinal scale) to unblinded (available to healthcare providers) monitor generated alarms in response to hypotension, tachycardia or hypoxemia. Myocardial injury after non-cardiac surgery (MINS) is defined by troponin elevation apparently from cardiac ischemia with or without signs and symptoms. It is a leading cause of postoperative mortality, with about 20% of all deaths being secondary to MINS.(6) About 80% of postoperative myocardial injury is clinically silent, detected only by troponin screening. MINS is strongly associated with both intraoperative (7, 8) and postoperative hypotension.(9, 10) However, nearly all MINS occurs during the initial three postoperative days suggesting that postoperative hypotension may be a major cause. As an exploratory outcome we will investigate whether the availability of continuous monitoring and interventions to reduce hypotension would decrease MINS. Assuming positive results, data from this pilot (N= 500 exposed to each of unblinded and blinded monitoring) trial will form the basis for a large randomized trial of ward monitoring assessing "hard" outcomes such as myocardial injury, respiratory failure, and death. In this context, unblinded monitoring will be continuous vital signs available at all times to healthcare providers, whereas blinded monitoring would be silenced monitoring with bedside providers having no access to continuous vital signs or associated alarms. Standard of care monitoring (every 4 hours vital signs checks) will be used as the comparator and will be available in blinded arm. Fail-safe alarms set at extreme limits will also be available in the blinded arm.

Specific Aim 1, primary: Determine whether continuous unblinded (vs. blinded) monitoring and nursing alerts reduce the duration of hypotension, tachycardia, or hypoxemia over the first 48 hours after surgery, without substantially increasing the duration of any of the 3 outcomes (i.e., noninferior on all, superior on at least one)

Hypothesis 1: Continuous unblinded postoperative blood pressure, heart rate, and saturation monitoring with associated nursing alerts and management strategies reduces the duration of hypotension (MAP <65 mmHg) and/or tachycardia (>90 beats/min) and/or desaturation (SpO2<90%). **Specific Aim 2, secondary**: Assess the effect of unblinded versus blinded continuous postoperative monitoring on the fraction of patients in which a clinical intervention is triggered due to hypotension (MAP <65 mmHg), tachycardia (>90 beats/min) or desaturation (SpO2<90%). Clinical nursing intervention responses over the duration of the trial will be categorized as (1) none,

(2) independent nursing intervention, (3) notification of physician team, or (4) activation of the hospital Emergency or Rapid Response System. These will also serve as surrogate markers of alarm fatigue for the bedside nurses. We will have at least two independent evaluators adjudicate monitor data and electronic medical records during the alarm period to define which of hypotension, tachycardia or hypoxemia was the primary trigger for each response. The main secondary outcome will be summarized for a patient as the worst clinical nursing intervention response among hypotension, tachycardia and desaturation, on the above ordinal scale. **Hypothesis 2**: Increasing bedside clinical nursing interventions (ranging from no response, to activation of the hospital rapid response team) are more common in patients randomized to unblinded monitoring and associated nursing alerts. **Specific Aim 3, exploratory**: Assess the effect of unblinded versus blinded continuous postoperative monitoring on the incidence of myocardial injury after non-cardiac surgery (MINS). **Hypothesis 3**: MINS is less common when continuous hemodynamic monitoring is available to clinicians.

Methods and Measures

Participant selection: We will include surgical inpatients (transferred to pre-designated wards) ≥65 years or ≥45 years with at least one cardiovascular risk factor (e.g. hypertension, diabetes) having noncardiac surgery who require a general or regional anesthesia and at least a 48 hours hospital stay after surgery. The proposed trial will receive approval by the Wake Forest Institutional Review Board. The risks of the proposed study are minimal since no patient will be denied routine monitoring and treatment as per institutional guidelines.

Protocol: Two wards at Wake Forest Baptist will be enrolled in a prospective ward- cluster randomized (to one of the two sequences) alternating cohort study,[27,28] where the wards alternate monitoring policies in 4-week blocks over the course of a calendar year, as outlined in Table 1. These two wards will be randomized to one of the two sequences displayed in Table 1 by a coin flip. Entire wards will be allocated to *continuous ward monitoring with alarms silenced and screens covered (blinded), or to continuous monitoring accessible to clinicians (unblinded) with pre-specified alerts at MAP <65 mmHg, heart rate >90 b/m, and* SpO_2 *<90%. Of note, to ensure patient safety and no risk in either arm, factory alarm limits (see table under human subjects protection) at extremes of physiological vital signs will stay on in the blinded/silenced arm, and both arms will have every 4 hourly checks by nursing teams.* The ViSi mobile is a portable wrist mounted (Sotera wireless, San Diego, CA) continuous ambulatory monitoring system functions throughout Wake Forest Baptist Health, and health care providers are very familiar with its use and interpretation of data. We will educate & collaborate with all of the relevant nursing teams, surgeons, resident doctors, and the Emergency Medical Response Team, regarding the trial protocol and expected responses. Attractive features of this study design include its ability to (i) be easily implemented within wards, as ward personnel won't have to keep track of subsets assigned to blinded and unblinded, (ii) protect against the Hawthorne effect, as each ward will be similarly exposed to unblinded monitoring,[29](iii) account for within-ward trends over time, e.g., personnel or procedure changes, as each ward will have similar amounts of exposure to unblinded monitoring, equally distributed over the course of a calendar year, and finally (iv) prevent contamination within ward as each patient will be only surrounded by patients with the same type of monitoring.

Table 1: Alternating cohort study design; U=unblinded, B=blinded to monitoring

	Weeks 1-4	Weeks 5-8	Weeks 9-12	Weeks 13-17	Weeks 18-21	Weeks 22-25	Weeks 26-29	Weeks 30-33	Weeks 34-37	Weeks 38-41	Weeks 42-44	Weeks 45-48	Weeks 49-52
Ward 1	U	B	U	B	U	B	U	B	U	B	U	B	U

Ward 2	B	U	B	U	B	U	B	U	B	U	B	U	B

Measurements: ViSi continuous monitoring will start on ward admission and continue until the 3rd postoperative morning or 48 continuous hours (whichever comes earlier) while patients remain hospitalized. The ViSi monitoring system is wrist-mounted and battery powered; it communicates via secure Wi-Fi. Values and alerts (in unblinded patients) will display on individual monitors and on each nursing station. *For blinded patients, nursing station screens will be turned off, and alarms disabled, except for fail-safe alarms at extreme limits and technical alerts.* (**specific aim 1**) *To ensure patient safety, all patients will also have routine intermittent nurse monitoring and any clinically appropriate interventions.* Details of interventions in response to unblinded alarms will be obtained from the electronic medical record and grouped on the pre-specified ordinal scale.(**specific aim 2**) Blood for troponin I will be obtained on the first three postoperative mornings; concentrations ≥0.03 ng/ml increase mortality by a factor-of-nine and will be considered MINS if there is no evidence of a non-ischemic etiology.[17](**specific aim 3**)

Setting

This study is taking place at Wake Forest Baptist Health-Winston Salem campus on post-surgical wards.

Subject selection criteria

We will include surgical inpatients (transferred to pre-designated wards) ≥65 years or ≥45 years with at least one cardiovascular risk factor (e.g. hypertension, diabetes) having noncardiac surgery who require a general or regional anesthesia and at least a 48 hours hospital stay after surgery. The proposed trial will receive approval by the Wake Forest Institutional Review Board.

- **Inclusion Criteria**
  >/= 65 years of age
  >/= 45 years of age with at least 1 cardiovascular risk (hypertension, diabetes)
  Requiring a general or regional anesthetic as part of their surgical procedure.
  Requiring (or anticipated to require) at least a 48 hour hospital stay after surgery.
- **Exclusion Criteria**
  < 48 hour hospital stay
  Receiving local anesthetic for their surgical procedure
  <65 years of age without a single cardiovascular risk factor or <45 years of age
- **Sample Size**
  The wards we plan to partner with at Wake Forest Baptist have approximately 2-4 new patient admissions per day. Given 365 days in a year, each ward expects to see about 365*3 = 1,095 patients per ward per year. We will exclude patients whose admissions occur at the end of a 4-week period and spill over into the subsequent 4-week period to avoid analyzing patients exposed to both blinded and unblinded monitoring. Assuming some level of ward transfer, spillover exclusions to subsequent 4-week periods, cost of monitoring, and patient refusal to wear the monitoring device, and use of age and risk based inclusion criteria cited earlier, we anticipate about n=500 participants in each ward over the course of the calendar year.

Interventions and Interactions

Two wards at Wake Forest Baptist will be enrolled in a prospective ward- cluster randomized (to one of the two sequences) alternating cohort study,[27,28] where the wards alternate monitoring policies in 4-week blocks over the course of a calendar year, as outlined in Table 1. These two wards will be randomized to one of the two sequences displayed in Table 1 by a coin flip. Entire wards will be allocated to *continuous ward monitoring with alarms silenced and screens covered (blinded), or to continuous monitoring accessible to clinicians (unblinded) with prespecified alerts at MAP <65 mmHg, heart rate >110 b/m, and* SpO_2 *<90%. Of note, to ensure patient safety and no risk in either arm, factory alarm limits (see table under human subjects protection) at extremes of physiological vital signs will stay on in the blinded/silenced arm, and both arms will have every 4 hourly checks by nursing teams.*

The 2 wards will alternate every 4 weeks (first Wednesday of each month changeover) as to blinded/unblinded allocations.

Outcome Measure(s)

- **Specific Aim 1, primary**: Determine whether continuous unblinded (vs. blinded) monitoring and nursing alerts reduce the duration of hypotension, tachycardia, or hypoxemia over the first 48 hours after surgery, without substantially increasing the duration of any of the 3 outcomes (i.e., noninferior on all, superior on at least one)

  Hypothesis 1: Continuous unblinded postoperative blood pressure, heart rate, and saturation monitoring with associated nursing alerts and management strategies reduces the duration of hypotension (MAP <65 mmHg) and/or tachycardia (>110 beats/min) and/or desaturation (SpO2<90%). **Specific Aim 2, secondary**: Assess the effect of unblinded versus blinded continuous postoperative monitoring on the fraction of patients in which a clinical intervention is triggered due to hypotension (MAP <65 mmHg), tachycardia (>110 beats/min) or desaturation (SpO2<90%). Clinical nursing intervention responses over the duration of the trial will be categorized as (1) none, (2) independent nursing intervention, (3) notification of physician team, or (4) activation of the hospital Emergency or Rapid Response System. These will also serve as surrogate markers of alarm fatigue for the bedside nurses. We will have at least two independent evaluators adjudicate monitor data and electronic medical records during the alarm period to define which of hypotension, tachycardia or hypoxemia was the primary trigger for each response. The main secondary outcome will be summarized for a patient as the worst clinical nursing intervention response among hypotension, tachycardia and desaturation, on the above ordinal scale.

  Hypothesis 2: Increasing bedside clinical nursing interventions (ranging from no response, to activation of the hospital rapid response team) are more common in patients randomized to unblinded continuous monitoring and associated nursing alerts. **Specific Aim 3, exploratory**: Assess the effect of unblinded versus blinded continuous postoperative monitoring on the incidence of myocardial injury after non-cardiac surgery (MINS). Blood for troponin I will be obtained on the first three postoperative mornings; concentrations ≥0.03 ng/ml increase mortality by a factor-of-nine and will be considered MINS if there is no evidence of a non-ischemic etiology.(11)

  Hypothesis 3: MINS is less common when continuous hemodynamic monitoring is available to clinicians and appropriate interventions are made in response to hypotension detected by the same.

  Ancillary outcomes: At the end of the study period and prior to discharge from the hospital, all included patients will be provided a research information document. This will include (via a link to a REDCap anonymous survey) a post study satisfaction questionnaire that will collect useful data on the patient's perception of continuous monitoring and the comfort/discomfort with the devices used in each arm. This

data will only be used with descriptive statistics and will be used to gain useful insight into the need for surveillance monitoring on hospital wards.

The following questions will be included in the post study survey questionnaire:

On a scale of 1-5 (1 = disagree 2 = somewhat disagree 3= neutral 4= somewhat agree and 5=agree), please rate your experience with vital signs checks during your hospital stay

1. Were continuous vital signs monitoring using a monitoring device important to you?
2. Was intermittent nursing checks (provider in the room) to check on your vital signs important to you?
3. Was the type of devices that were used to monitor your vital signs important to you?
4. Were you inconvenienced with the portable monitoring device on your wrist?
5. Were you inconvenienced with the intermittent nursing checks (provider in the room) to check on your vital signs?
6. Did alarms inconvenience you?
7. Would you feel safer with a continuous monitoring device strapped to you all the time while you recover in the hospital?
8. Is the size of the continuous monitoring device important for you?
9. Is the ability to ambulate/walk around freely while being monitored important for you?
10. Would it be a good idea to continue to monitor your vital signs remotely even after you go home?

Analytical Plan

Both ward and individual level data will be collected for statistical analysis. The two wards will be compared on baseline characteristics using descriptive statistics. Variables with an absolute standardized difference > 0.10 will be considered imbalanced and will be adjusted for in all analyses. Analyses will be done under the modified intention-to-treat principle, where enrolled patients will be included in all analyses as long as they received at least some of blinded or unblinded ward monitoring. Missing data as well as the reason for missing will be summarized. Our modeling framework (generalized linear mixed effects modeling) makes the same assumptions about missing data as multiple imputation.

Data Storage Sotera is responsible for the ViSi monitoring data, which will be stored in the Sotera cloudbased database. This data set will contain device IDs, along with dates, to be merged to EPIC charts collected here at Wake Forest School of Medicine. We have easily piloted this data collection approach from the period February 2015 to April 2018, and these merged data now live in a SQL databased here at Wake. Dr. Amit Saha, PhD., Assistant Professor of Anesthesiology and member of the Wake Forest Center for Biomedical Informatics will provide expertise on data collection and storage.

Sample size and power: As is standard in the clustered study design literature, [30]we will perform a power calculation that (i) assumes individual-level randomization and then (ii) adjusts for cluster-level correlation, as study participants cannot be viewed as statistically independent within a cluster and are not actually individually randomized. Presumably, for **Aim 1**, the true incidence of hypotension and tachycardia, based on unblinded continuous monitoring, will be much higher than that measured via blinded routine monitoring. We have observed that median [quartiles] minutes under MAP of 65 mmHg of 6 [0, 19] or higher for noncardiac patients who suffer AKI and MI. In our power calculation to follow, we assume that the unblinded patients will have approximately mean 7.3 minutes exposed to MAP <65 mmHg with standard deviation of 13, whereas blinded patients will have mean 13 minutes exposed to MAP <65 mmHg with a standard deviation of 13.

In a two-group individually randomized study, a two-sample two-sided t-test with type 1 error set to 0.05 would have 100% power to reject the null hypothesis that the exposed time to MAP <65 mmHg was the same in unblinded versus blinded under the alternative hypothesis listed above. With our anticipated

effect size, we would have 80% power in an individually randomized study for sample sizes as low as 89 per group. Given the clustered nature of our study design, we must adjust our calculations by the design effect, $D_{eff} = 1+(m-1)*ICC$, where m is the number of patients per ward in a given month and ICC $\in [0, 1]$ is the correlation of patients within a ward. The figure below displays how sample size to achieve 80% statistical power for our anticipated effect size will inflate as a function of increasing ICC.

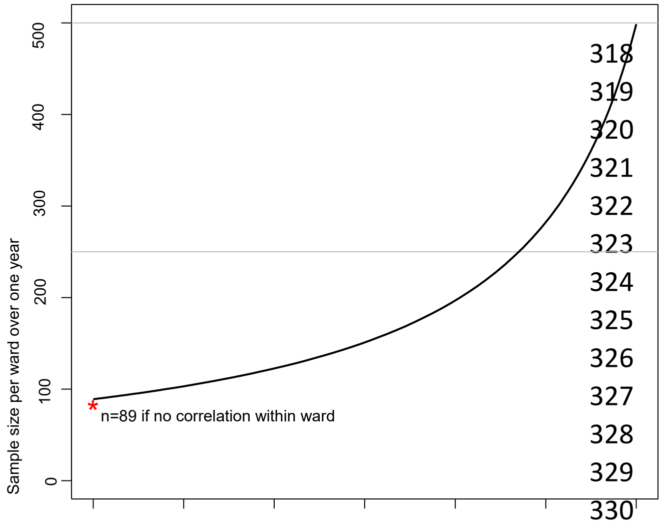

The wards we plan to partner with at Wake Forest Baptist have approximately 2-4 new patient admissions per day. **Given 365 days in a year, each ward expects to see about** 365*3 = *1,095 patients per ward per year*. We will exclude patients whose admissions occur at the end of a 4-week period and spill over into the subsequent 4-week period to avoid analyzing patients exposed to both blinded and unblinded monitoring. Assuming some level of ward transfer, spillover exclusions to subsequent 4week periods, cost of monitoring, and patient refusal to participate in the study, and use of age and risk based inclusion criteria cited earlier, we anticipate about *n=500 participants in each ward over the course of the calendar year*. As seen in Table 1, these 500 participants per ward will be somewhat evenly split into m=13 distinct groups of approximately 500/13

= 39 patients. Our study design is similar to a clusterrandomized study that compares 13 groups of 39 pati

randomized to unblinded monitoring to 13 groups of 39 patients randomized to blinded monitoring. As shown in the above figure, we will have sufficient power as long as (i) ICC remains below 0.12, which is a reasonable assumption,[26] and we can recruit upwards of n=500 per ward over the course of a year. If we plan to only recruit n=300 per ward, ICC could be up to 0.10.

Statistical Analysis Plan

Aim 1. We will assess the effect of unblinded continuous monitoring and the associated alerts on the cumulative duration (min/hour) of each of hypotension, tachycardia, and desaturation using negative binomial regression, within a generalized linear mixed effects modeling framework (to adjust for correlation of participants within ward with a random effect for ward). Individuals exposed to unblinded versus blinded monitoring will be compared on the number of minutes less than each threshold (i.e., a count outcome), considering the total number of minutes observed for the patient as an offset. Treatment effects will be reported as the estimated incidence rate ratio of unblinded to blinded and confidence interval separately for hypotension, tachycardia, and hypoxia. A rate ratio less than 1 will indicate a favorable effect of unblinded monitoring on the outcome, i.e., participants are less likely to be exposed to harmful conditions, e.g., MAP<65, in the presence of unblinded monitoring.

Aim 2. We will assess the effect of continuous monitoring and associated alerts on the ordinal clinical intervention outcome which measures the single most extreme/elevated clinical intervention incurred by a patient for any of the 3 outcome variables of interest (hypotension, tachycardia, hypoxia) during the study period, summarized on an ordinal scale as (1) none, (2) independent nursing intervention, (3) notification of physician team, or (4) activation of the hospital Emergency or Rapid Response system using ordinal logistic regression model (with a random effect for ward) and expressing the treatment effect as the odds ratio and confidence interval for having a more extreme/elevated clinical intervention in the unblinded versus blinded monitoring. As in Aim 1, an odds ratio less than 1 will indicate a favorable effect of unblinded monitoring. **Aim 3.** We will assess the treatment effect on MINS (yes/no) during the study period using logistic regression with a random effect for ward. Results for Aims 2 and 3 are exploratory and will be used mainly for hypothesis generation for future studies. In this pilot study we will use a significance level of 0.05 for each outcome variable of interest, making no correction for assessing multiple outcome variables. As in previous Aims, an odds ratio less than 1 will indicate a favorable effect of unblinded monitoring

Results will be analyzed initially using descriptive statistics. Comparison between groups will be done using chi square tests for proportions, and t-tests or ANOVA procedures for continuous variables. Regression analysis will be performed to identify independent outcome predictors. Other inferential statistical analysis will be conducted as appropriate.

Addition of Interim Analysis

Our initial sample size calculations were powered to detect an effect size of a 5.7-minute difference between treatment groups in terms of time exposed to MAP < 65mmHG (assuming an equal standard deviation of 13 minutes exposed for each group). We calculated our sample size projections to have 80% power with a 5% type-I error rate. In our calculations, we recognized there currently exists a degree of uncertainty regarding intra-ward correlation (ICC), the degree of which has implications on our necessary sample size. As ICC increases, our required sample size to achieve 80% power increases. To address this uncertainty, we propose conducting an interim analysis at 6-months to assess the estimated ICC of our population, from which we can adjust our period of accrual accordingly to ensure adequate power without unnecessary (i.e. prolonged) recruitment. In this interim analysis, we will not evaluate our primary hypothesis assessing a difference between groups with respect to our primary outcomes. Therefore, we do

not propose amending our primary statistical analysis plan to account for multiple looks. At 6 months, we expect to have accrued approximately 250 patients. The decision rules based on our interim assessment of ICC are as follows:

- If the ICC is sufficiently low enough to have achieved ≥80% power given our existing sample size at the time of the interim analysis, we will conclude accrual at the end of the current (ongoing) 4-week phase of the study. Given the timeline of the study, this would mean our interim analysis of ICC would be conducted at week 26 of the study, and thus the study would conclude after week 29 (the 4th week of the study phase accruing over weeks 26-29). - If the estimated ICC is higher than what would yield ≥80% power at the time of the interim assessment, but less than our maximum assumed possible ICC of 0.12 requiring 500 patients, we will re-estimate our required sample size assuming the estimated ICC from the first 6 months. Based on our new estimate for required sample size, we will adjust our timeframe for accrual accordingly such to complete the study after the month in which we expect to meet our necessary sample size.
- If the estimated ICC > 0.12, thus requiring > 52 weeks of accrual, we will estimate the amount of additional time necessary to reach our required sample size, beyond our initially proposed 52 weeks. Our decisions to continue or stop the study due to futility will be made based on funds and time available.

Approach To Potential Limitations

(i)Although the ViSi monitor is lightweight and battery powered, patients may refuse to be tethered to wear the system for 48 hours. We are currently using this monitor, with good patient cooperation; specifically, we have been able to obtain reliable near-continuous recordings in postoperative patients over several postoperative days. The ViSi Mobile technology is cleared by the FDA and is accurate within ±8 mmHg (SD) against a radial arterial catheter.

(ii) Continuous monitoring may detect more hemodynamic and respiratory critical events than routine nurse monitoring at four-hour intervals. This *per se* may not prompt nurses and clinicians to intervene — much less that the interventions will prove effective. We will provide a broad framework of suggested interventions, none of which would be binding. We will also evaluate the frequency and nature of interventions. Since, alarm fatigue from the continuously generated alarms (some of which may be false) may be common, we propose to categorize nursing responses on an incremental scale from 1-4. From these data, it will be apparent whether nurses responded quickly to the alarms, and with reasonable interventions either themselves or from the physician alerted, or via rapid response team intervention. This data will also over the time course of the study tell us an estimate of alarm fatigue. (iii) In addition, our primary outcome characterizes important mechanisms by which hemodynamic perturbations and desaturation might contribute to myocardial injury after noncardiac surgery (MINS) and serious respiratory complications. This study will be underpowered for an exploratory outcome of MINS, but the pilot data will be used to adequately power the proposed R01 which will be a much larger trial.

Human Subjects Protection

Subject Recruitment Methods

With collaboration with nursing management, 2 postsurgical units will be identified who currently have the capabilities to utilize ViSi monitoring. The surgical population that these 2 units receive from the operative area will likely include a majority of general surgery, gynecological, urological, orthopedic, spine and plastic surgery. Surgical leaders with each of these specialties have been approached and will be re-approached prior to the study to ascertain their willingness to participate. In case, a specific surgical team feels that a certain patient should not be included in the proposed study that patient will be withdrawn and likely moved to another postsurgical ward in the hospital.

Informed Consent

A waiver of the requirements for **signed** informed consent and a waiver of HIPPA authorization is being requested. This research presents no more than minimal risk of harm to subjects and involves no procedures which written consent is normally required outside of the research context. This device is currently not utilized routinely on all hospital units at this time. The risks of the proposed study are minimal since no patient will be denied routine monitoring and treatment as per institutional guidelines. We will however provide every patient with a research information sheet before they leave the hospital as part of their after visit summary. This sheet will provide all necessary details about the data that has been collected and how it will likely be used for research. Every patient will be given the opportunity to opt out of their data being used for the study. This document will include (via a link to a REDCap anonymous survey) a post study satisfaction questionnaire that will collect useful data on the patient's perception of continuous monitoring and the comfort/discomfort with the devices used in each arm. (See questions and details under ancillary outcomes – under specific outcomes)

Safety considerations

As previously specified, this study poses minimal risk in either of the randomized clusters. Both the randomized clusters will continue to receive at least every four hourly vital signs checks, which is current standard of care across the nation. Both arms (including the blinded arm) will always have factory alarm limits (as specified in the table below) always switched on, so no critical event is missed on any patient (no matter what cluster that patient is part of). Therefore though one cluster of patients will have continuous monitoring silenced and blinded, extreme alarms limits will always be available and standard of care intermittent monitoring will also always be available on these patients.

Vital Sign	Configuration Options (Factory Limit Parameters)		Annunciation Delay (seconds)	Sotera Recommended Limits	Hospital Selection
Critical Low HR	Upper	N/A	5	N/A	NA
	Lower	18		18	18
Heart Rate	Upper	200	15	150	150
	Lower	30		30	35
Pulse Rate	Upper	200	60	150	150
	Lower	30		30	35
BP Systolic	Upper	240	240	190	200
	Lower	70		Off	80
BP Diastolic	Upper	170	120	Off	OFF
	Lower	60		Off	OFF
MAP	Upper	170	60	Off	OFF
	Lower	58		60	OFF
Respiration	Upper	40	120	35	35
	Lower	4		4	4
SpO2	Upper	N/A	90	N/A	NA
	Lower	80		85	85

Confidentiality and Privacy

Confidentiality will be protected by collecting only information needed to assess study outcomes, minimizing to the fullest extent possible the collection of any information that could directly identify subjects, and maintaining all study information in a secure manner. To help ensure subject privacy and confidentiality, only a unique study identifier will appear on the data collection form. Any collected patient identifying information corresponding to the unique study identifier will be maintained on a linkage file, store separately from the data. The linkage file will be kept secure, with access limited to designated study personnel. Following data collection subject identifying information will be destroyed following data analysis, consistent with data validation and study design, producing an anonymous analytical data set. Data access will be limited to study staff. Data and records will be kept locked and secured, with any computer data password protected. No reference to any individual participant will appear in reports, presentations, or publications that may arise from the study.

Data and Safety Monitoring

The principal investigator will be responsible for the overall monitoring of the data and safety of study participants. The principal investigator will be assisted by other members of the study staff.

Mentorship and role of other key investigators: Dr. Khanna will oversee all aspects of the study including IRB approval, pre-study education and orientation of nursing and research teams, patient recruitment, enrollment, monitoring, and data analysis. Dr. Khanna will collaborate with the monitoring device manufacturers (Sotera wireless) to ensure quality of data collection. Dr. Walter Ambrosius (professor and chair biostatics and public health sciences) initially supervised the creation of trial statistical methodology and will mentor Dr. Khanna during data analysis and for the construct of a robust plan for analysis on a follow up grant mechanism. Drs. Eric Kirkendall (chief for the center for healthcare innovation), Dr. Brian Wells (director for the center for biomedical informatics), and Dr. Daniel Sessler (professor & chair, department of outcomes research at Cleveland Clinic) will guide Dr. Khanna in development of a best mechanistic process for data collection, and interpretation/presentation of the results, and will provide direct mentorship for the proposed R01. Given their expertise in clinical informatics and innovation, Drs. Kirkendall and Wells, are well positioned to assist Dr. Khanna with testing of the data set, (including future device/technology innovations and utilization for predictive analytics) as well as troubleshooting any issues that may arise with the current implementation of this monitoring technology. The entire mentoring team will also participate and guide Dr. Khanna in data analysis, interpretation, dissemination (abstract and manuscript review), and NIH R01 application preparedness.
In addition, this study is appropriately supported by the nursing leadership at WFBMC, including the CNO Dr. Deb Harding, Director of Nursing Research, Dr. Carolyn Huffman, Nursing Informatics, Ann Faris & Robin Hack, amongst others. Physician leaders include Dr. Robert Weller, Professor of Anesthesiology, who has championed use of the ViSi device across WFBMC for the last 4 years, and the chairs of the CIIRRC group Dr(s). Chad Miller and Clark Files. Furthermore, additional support with data analysis is also being provided by Dr. Joseph Rigdon from the School of Public Health Sciences. Research support is also provided by the department of Anesthesiology, including data handling support by Dr. Amit Saha (departmental data scientist) and by two senior research nurses along with 6-8 research assistants, each of whom will be appropriately trained prior to be being introduced into the trial.

Reporting of Unanticipated Problems, Adverse Events or Deviations

Any unanticipated problems, serious and unexpected adverse events, deviations or protocol changes will be promptly reported by the principal investigator or designated member of the research team to the IRB and sponsor or appropriate government agency if appropriate.

SUBSTUDY

In an effort to determine the nursing perceptions of the benefits and failures of continuous hemodynamic monitoring systems as well as the value of these devices in the care of their patients, we propose a sub-study to evaluate on both of the stated research units (9 Ardmore East and 10 Reynolds Tower) and the non-research units who have continuous hemodynamic

monitoring utilizing the ViSi monitoring system. These surveys are identical, will be anonymous and unidentifiable, and will be distributed manually by research staff at various timepoints in an effort to capture the most responses. Information to be collected include the following types of information: the importance of continuous monitoring important to nursing practice and to the safety of their patients, perceptions of increased workload based on the alerts received, accuracy in alerting to patient deterioration, appropriateness of the alarm limits, alarms that cause the most concern when received, and how the nurse perceives the ease of use and confidence in the ViSi monitoring system. We are capturing the role of the nursing staff on that unit, unit worked on and number of years in practice.

We feel the information received on the conduct of this survey will provide feedback to the clinical research team and the clinical practice team practitioners with much needed information in order to see and identify barriers to the implementation of safe and effective continuous monitoring systems throughout the hospital. This is specifically important because the successful use of a continuous monitoring system and associated alarms is dependent on the actual response to alarms with actions that prevent harm from happening or further escalating.

We are asking for a waiver of informed consent for this survey distribution. A waiver of signed consent is being asked. The survey questions are evaluating nurses experiences pertaining to their positions. Information concerning the purpose of the surveys and assurance of confidentiality and no retaliation for responses. No signature would be obtained as the only record linking the subject and the research would be the informed consent form and the principal risk would be potential harm resulting from a breach of confidentiality.

References

1. Bartels K, Karhausen J, Clambey ET, Grenz A, Eltzschig HK: Perioperative organ injury. Anesthesiology 2013; 119: 1474-89

2. de Vries EN, Ramrattan MA, Smorenburg SM, Gouma DJ, Boermeester MA: The incidence and nature of in-hospital adverse events: a systematic review. Qual Saf Health Care 2008; 17: 216-23

3. Perman SM, Stanton E, Soar J, Berg RA, Donnino MW, Mikkelsen ME, Edelson DP, Churpek MM, Yang L, Merchant RM, American Heart Association's Get With the Guidelines-Resuscitation I: Location of In-Hospital Cardiac Arrest in the United States-Variability in Event Rate and Outcomes. J Am Heart Assoc 2016; 5

4. Andersen LW, Berg KM, Chase M, Cocchi MN, Massaro J, Donnino MW, American Heart Association's Get With The Guidelines-Resuscitation I: Acute respiratory compromise on inpatient wards in the United States: Incidence, outcomes, and factors associated with in-hospital mortality. Resuscitation 2016; 105: 123-9

5. Lyons PG, Edelson DP, Carey KA, Twu NM, Chan PS, Peberdy MA, Praestgaard A, Churpek MM, American Heart Association's Get With the Guidelines - Resuscitation I: Characteristics of Rapid Response Calls in the United States: An Analysis of the First 402,023 Adult Cases From the Get With the Guidelines Resuscitation-Medical Emergency Team Registry. Crit Care Med 2019; 47: 1283-1289

6. Devereaux PJ, Sessler DI: Cardiac complications in patients undergoing major noncardiac surgery. N Engl J Med 2015; 373: 2258-2269

7. Walsh M, Devereaux PJ, Garg AX, Kurz A, Turan A, Rodseth RN, Cywinski J, Thabane L, Sessler DI: Relationship between intraoperative mean arterial pressure and clinical outcomes after noncardiac surgery: Toward an empirical definition of hypotension. Anesthesiology 2013; 119: 507-515

8. Mascha EJ, Yang D, Weiss S, Sessler DI: Intraoperative mean arterial pressure variability and 30day mortality in patients having noncardiac surgery. Anesthesiology 2015; 123: 79-91

9. Devereaux PJ, Yang H, Yusuf S, Guyatt G, Leslie K, Villar JC, Xavier D, Chrolavicius S, Greenspan L, Pogue J, Pais P, Liu L, Xu S, Malaga G, Avezum A, Chan M, Montori VM, Jacka M, Choi P: Effects of extended-release metoprolol succinate in patients undergoing non-cardiac surgery (POISE trial): a randomised controlled trial. Lancet 2008; 371: 1839-47

10. Devereaux PJ, Sessler DI, Leslie K, Kurz A, Mrkobrada M, Alonso-Coello P, Villar JC, Sigamani A, Biccard BM, Meyhoff CS, Parlow JL, Guyatt G, Robinson A, Garg AX, Rodseth RN, Botto F, Lurati Buse G, Xavier D, Chan MT, Tiboni M, Cook D, Kumar PA, Forget P, Malaga G, Fleischmann E, Amir M, Eikelboom J, Mizera R, Torres D, Wang CY, Vanhelder T, Paniagua P, Berwanger O, Srinathan S, Graham M, Pasin L, Le Manach Y, Gao P, Pogue J, Whitlock R, Lamy A, Kearon C, Chow C, Pettit S, Chrolavicius S, Yusuf S, Poise-2 Investigators: Clonidine in patients undergoing noncardiac surgery. N Engl J Med 2014; 370: 1504-13

11. Sun Z, Sessler DI, Dalton JE, Devereaux PJ, Shahinyan A, Naylor AJ, Hutcherson MT, Finnegan PS, Tandon V, Darvish-Kazem S, Chugh S, Alzayer H, Kurz A: Postoperative hypoxemia is common and persistent: A prospective blinded observational study. Anesth Analg 2015; 121: 709-715

12. Chung F, Subramanyam R, Liao P, Sasaki E, Shapiro C, Sun Y: High STOP-Bang score indicates a high probability of obstructive sleep apnoea. Br J Anaesth 2012; 108: 768-75

13. Khanna AK, Sessler DI, Sun Z, Naylor AJ, You J, Hesler BD, Kurz A, Devereaux PJ, Saager L: Using the STOP-BANG questionnaire to predict hypoxaemia in patients recovering from noncardiac surgery: a prospective cohort analysis. Br J Anaesth 2016; 116: 632-40

14. Belcher AW, Khanna AK, Leung S, Naylor AJ, Hutcherson MT, Nguyen BM, Makarova N, Sessler DI, Devereaux PJ, Saager L: Long-acting patient-controlled opioids are not associated with more postoperative hypoxemia than short-acting patient-controlled opioids after noncardiac surgery: A cohort analysis. Anesth Analg 2016; 123: 1471-1479

15. Hopf HW: Preventing Opioid-Induced Postoperative Hypoxemia: No Simple Answer? Anesth Analg 2016; 123: 1356-1358

16. The Vascular Events In Noncardiac Surgery Patients Cohort Evaluation (VISION) Study Investigators: Association between postoperative troponin levels and 30-day mortality among patients undergoing noncardiac surgery. JAMA 2012; 307: 2295-304

17. The Vascular events In noncardiac Surgery patIents cOhort evaluatioN (VISION) Investigators: Myocardial injury after noncardiac surgery: A large, international, prospective cohort study establishing diagnostic criteria, characteristics, predictors, and 30-day outcomes. Anesthesiology 2014; 120: 564-78

18. Sessler DI, Meyhoff CS, Zimmerman NM, Mao G, Leslie K, Vasquez SM, Balaji P, Alvarez-Garcia J, Cavalcanti AB, Parlow JL, Rahate PV, Seeberger MD, Gossetti B, Walker SA, Premchand RK, Dahl RM, Duceppe E, Rodseth R, Botto F, Devereaux PJ: Period-dependent Associations between Hypotension During and for 4 Days after Noncardiac Surgery and a Composite of Myocardial Infarction and Death: A Substudy of the POISE-2 Trial. Anesthesiology 2017

19. Turan A, Chang C, Cohen B, Saasouh W, Essber H, Yang D, Ma C, Hovsepyan K, Khanna AK, Vitale J, Shah A, Ruetzler K, Maheshwari K, Sessler DI: Incidence, Severity, and Detection of Blood Pressure Perturbations after Abdominal Surgery: A Prospective Blinded Observational Study. Anesthesiology 2019; 130: 550-559

20. Ruetzler K, Yilmaz HO, Turan A, Zimmerman NM, Mao G, Hung MH, Kurz A, Sessler DI: Intraoperative tachycardia is not associated with a composite of myocardial injury and mortality after noncardiac surgery: A retrospective cohort analysis. Eur J Anaesthesiol 2019; 36: 105-113
21. Khanna AK, Overdyk FJ, Greening C, Di Stefano P, Buhre WF: Respiratory depression in low acuity hospital settings-Seeking answers from the PRODIGY trial. J Crit Care 2018; 47: 80-87
22. Lee LA, Caplan RA, Stephens LS, Posner KL, Terman GW, Voepel-Lewis T, Domino KB: Postoperative opioid-induced respiratory depression: a closed claims analysis. Anesthesiology 2015; 122: 659-65
23. Khanna AK, Ahuja S, Weller RS, Harwood TN: Postoperative ward monitoring - Why and what now? Best Pract Res Clin Anaesthesiol 2019; 33: 229-245
24. Khanna AK, Hoppe P, Saugel B: Automated continuous noninvasive ward monitoring: future directions and challenges. Crit Care 2019; 23: 194
25. Ayad S, Khanna AK, Iqbal SU, Singla N: Characterisation and monitoring of postoperative respiratory depression: current approaches and future considerations. Br J Anaesth 2019; 123: 378-391
26. Adams G, Gulliford MC, Ukoumunne OC, Eldridge S, Chinn S, Campbell MJ: Patterns of intracluster correlation from primary care research to inform study design and analysis. J Clin Epidemiol 2004; 57: 785-94
27. Maheshwari K, Ahuja S, Mascha EJ, Cummings KC, 3rd, Chahar P, Elsharkawy H, Kurz A, Turan A, Sessler DI: Effect of Sevoflurane Versus Isoflurane on Emergence Time and Postanesthesia Care Unit Length of Stay: An Alternating Intervention Trial. Anesth Analg 2019
28. Kopyeva T, Sessler DI, Weiss S, Dalton JE, Mascha EJ, Lee JH, Kiran RP, Udeh B, Kurz A: Effects of volatile anesthetic choice on hospital length-of-stay: a retrospective study and a prospective trial. Anesthesiology 2013; 119: 61-70
29. McCarney R, Warner J, Iliffe S, van Haselen R, Griffin M, Fisher P: The Hawthorne Effect: a randomised, controlled trial. BMC Med Res Methodol 2007; 7: 30
30. Hemming K, Girling AJ, Sitch AJ, Marsh J, Lilford RJ: Sample size calculations for cluster randomised controlled trials with a fixed number of clusters. BMC Med Res Methodol 2011; 11: 102
