# Supplementary material for: Continuous vs Intermittent Postoperative Vital Sign Monitoring: A Cluster Randomized Crossover Trial
Source: JAMA Netw Open. 2026 Mar 26;9(3):e263290. doi: 10.1001/jamanetworkopen.2026.3290 (PMC13022735; doi:10.1001/jamanetworkopen.2026.3290)
Supplement: Supplement 2. — eFigure 1. Percentage of Patients With Time Beyond an SpO2 <90% eFigure 2. Percentage of Patients With Time Beyond a MAP <65 mm Hg eFigure 3. Percentage of Patients With Time Beyond a Heart Rate >110/Minute eFigure 4. Percentage of Patients With Time Beyond a Heart Rate <50/Minute eFigure 5. Percentage of Patients With Time Beyond a MAP <70 mm Hg eFigure 6. Percentage of Patients With Time Beyond a MAP <75 mm Hg eFigure 7. Percentage of Patients With Time Beyond a MAP <80 mm Hg eFigure 8. Percentage of Patients With Time Beyond a MAP >130 mm Hg eFigure 9. Adjusted Relative Risk Ratios of Intervention in Continuous Monitoring Relative to Intermittent Monitoring eTable 1. Schematic for Planned Alternating Interventions Trial eTable 2. Actual Days and Patients in Each Cluster of the Alternating Interventions eTable 3. Frequency of Unfiltered Alarms During the Trial Period for the Set Thresholds eTable 4. Troponin and Myocardial Injury After Noncardiac Surgery (MINS) [file jamanetwopen-e263290-s002.pdf]

Supplemental Online Content

Khanna AK, O'Connell NS, Saha AK, et al. Continuous vs intermittent postoperative vital sign monitoring. *JAMA Netw Open.* 2026;9(3):e263290. doi:10.1001/jamanetworkopen.2026.3290

eFigure 1. Percentage of Patients With Time Beyond an SpO_2 <90%

eFigure 2. Percentage of Patients With Time Beyond a MAP <65 mm Hg

eFigure 3. Percentage of Patients With Time Beyond a Heart Rate >110/Minute

eFigure 4. Percentage of Patients With Time Beyond a Heart Rate <50/Minute

eFigure 5. Percentage of Patients With Time Beyond a MAP <70 mm Hg

eFigure 6. Percentage of Patients With Time Beyond a MAP <75 mm Hg

eFigure 7. Percentage of Patients With Time Beyond a MAP <80 mm Hg

eFigure 8. Percentage of Patients With Time Beyond a MAP >130 mm Hg

eFigure 9. Adjusted Relative Risk Ratios of Intervention in Continuous Monitoring Relative to Intermittent Monitoring

eTable 1. Schematic for Planned Alternating Interventions Trial

eTable 2. Actual Days and Patients in Each Cluster of the Alternating Interventions

eTable 3. Frequency of Unfiltered Alarms During the Trial Period for the Set Thresholds

eTable 4. Troponin and Myocardial Injury After Noncardiac Surgery (MINS)

This supplemental material has been provided by the authors to give readers additional information about their work.

© 2026 Khanna AK et al. *JAMA Network Open*.

eFigure1 : Percentage of patients with time beyond an SpO2 <90%

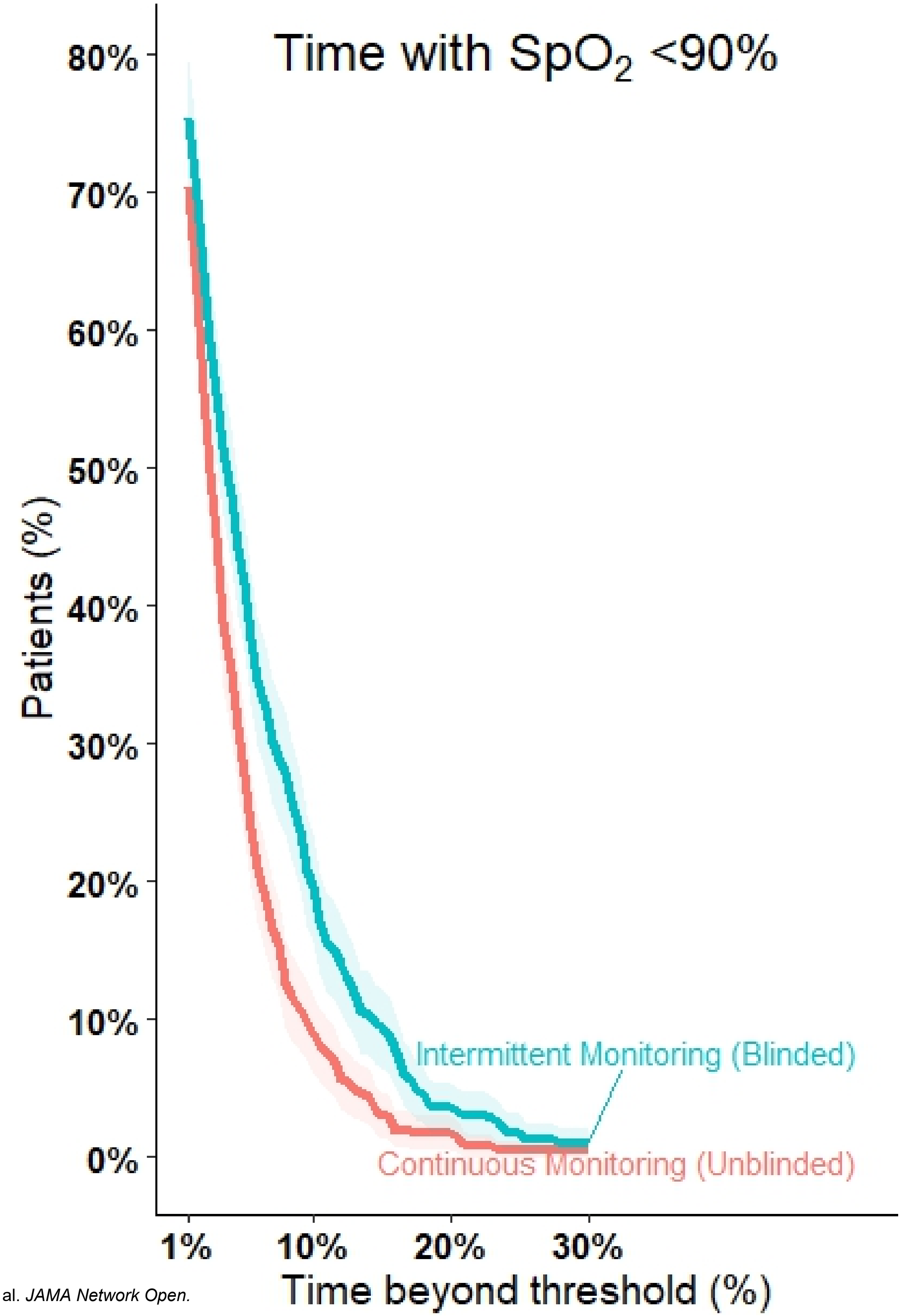

© 2026 Khanna AK et al. *JAMA Network Open.*

eFigure 2 : Percentage of patients with time beyond a MAP <65 mmHg

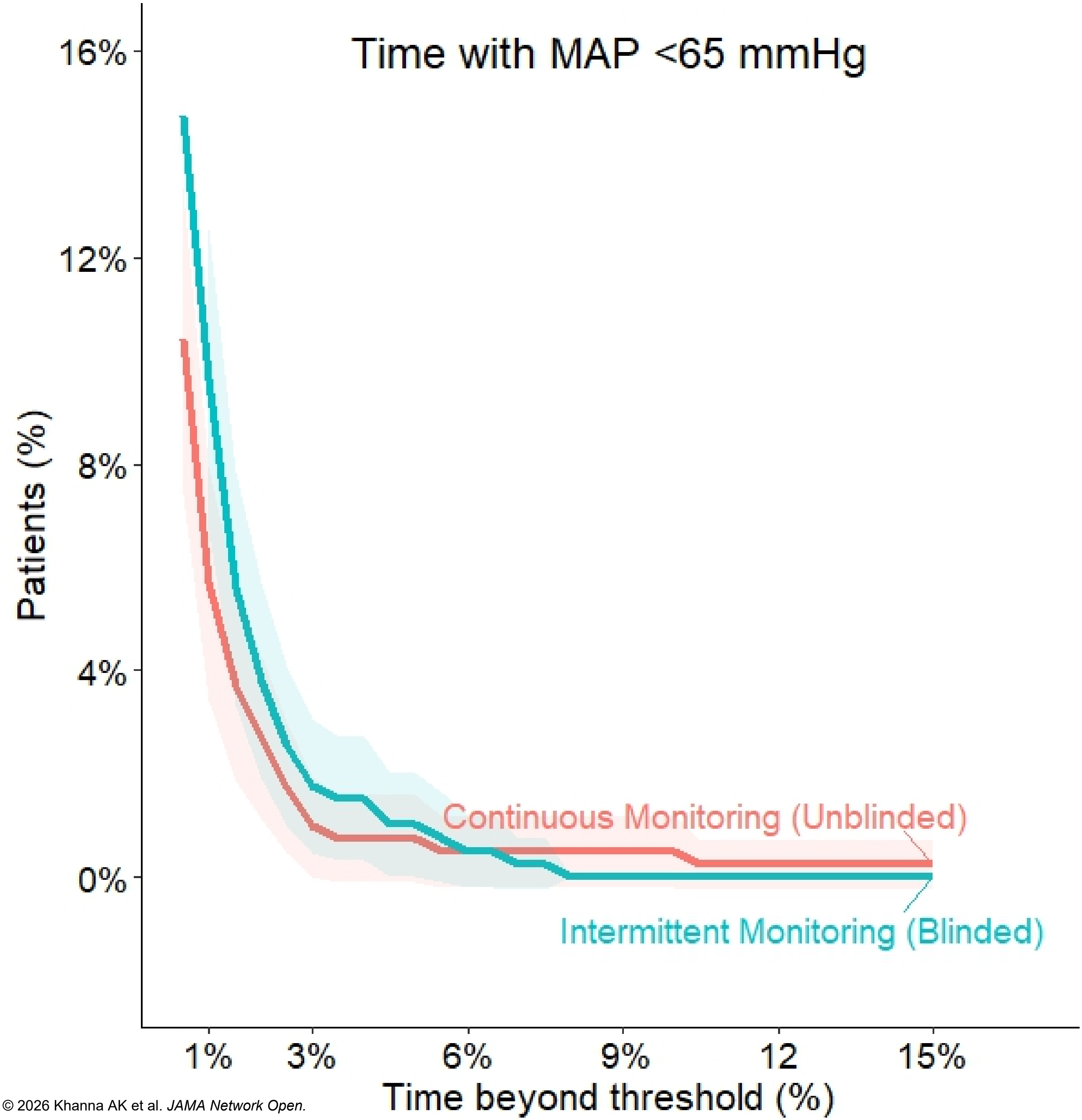

© 2026 Khanna AK et al. *JAMA Network Open.*

eFigure 3 : Percentage of patients with time beyond a HR > 110/minute

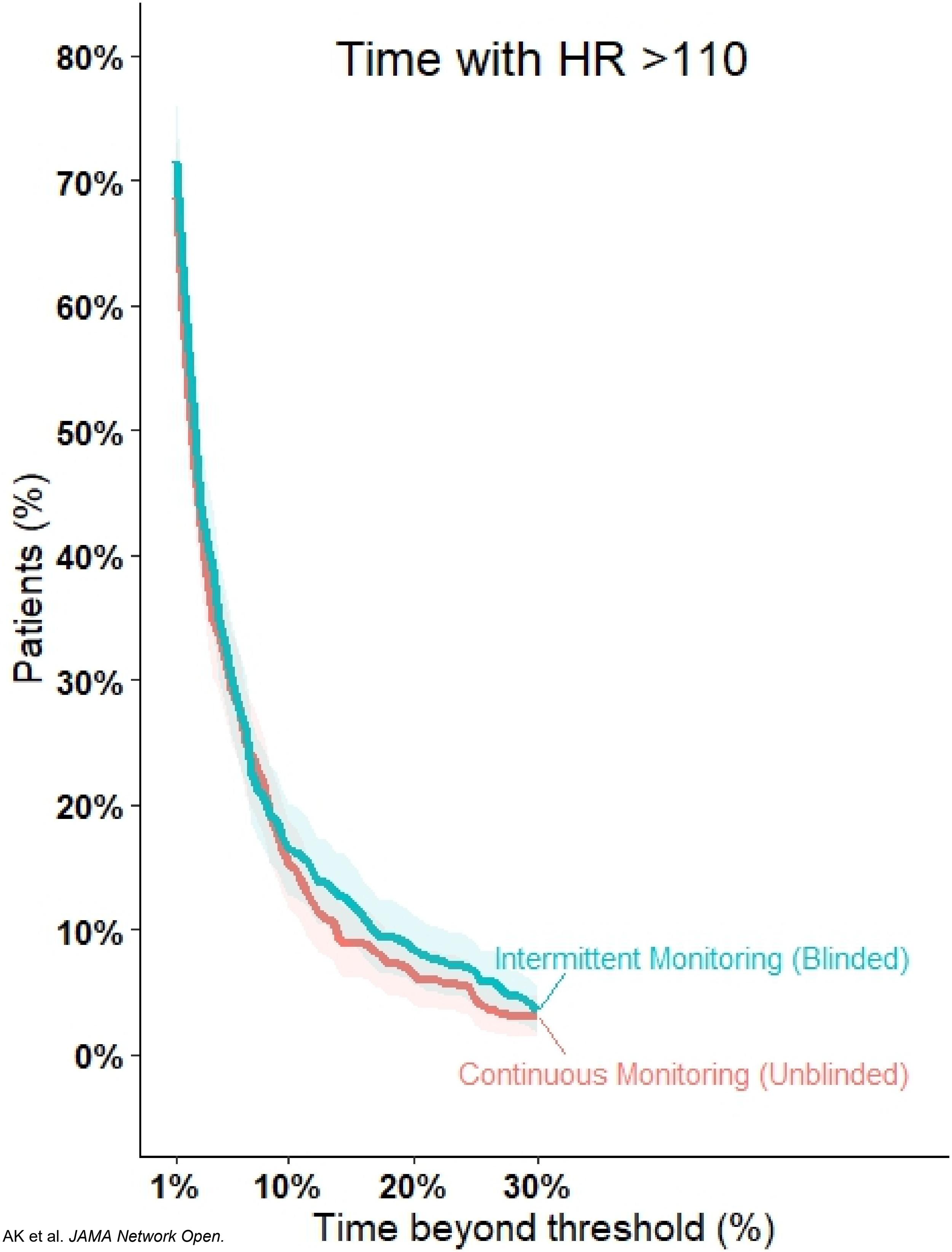

© 2026 Khanna AK et al. *JAMA Network Open*.

eFigure 4 : Percentage of patients with time beyond a HR <50/minute

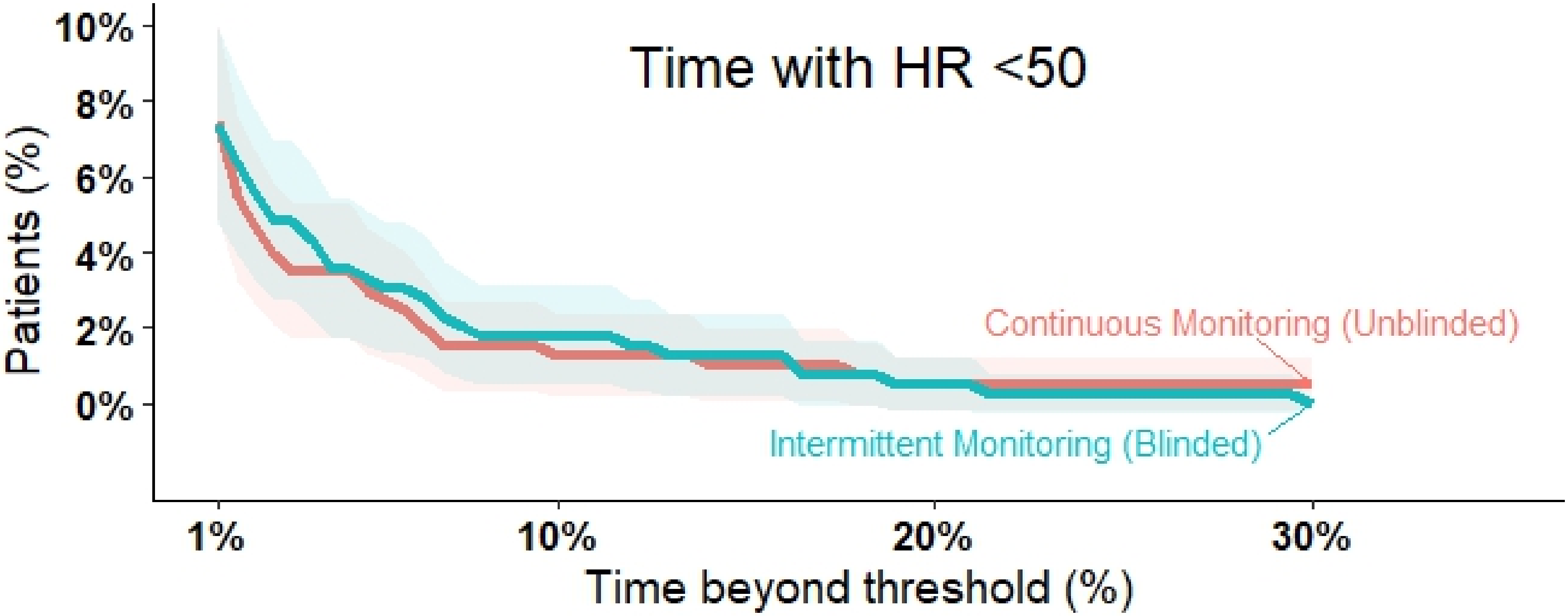

© 2026 Khanna AK et al. *JAMA Network Open.*

eFigure 5 : Percentage of patients with time beyond a MAP <70 mmHg

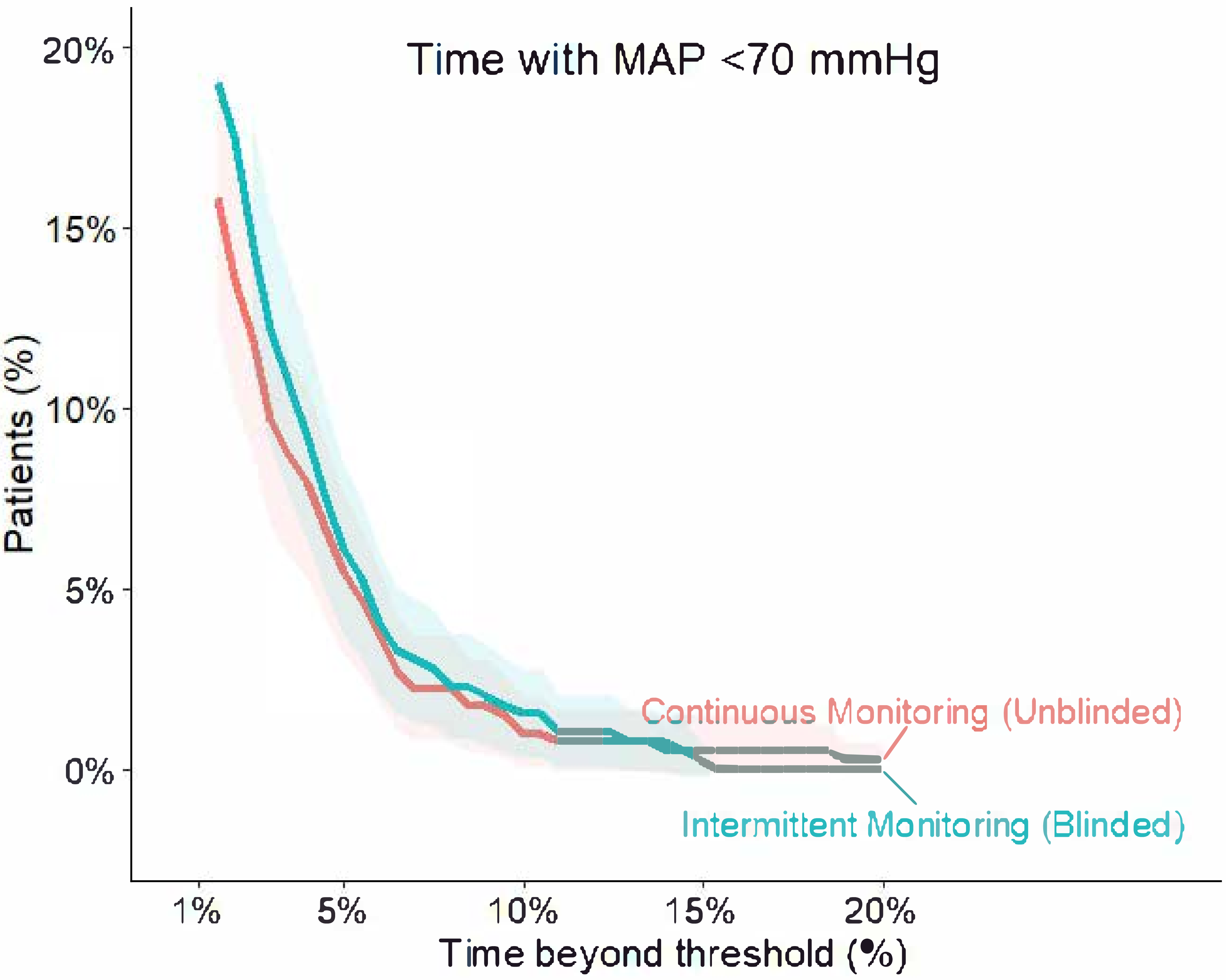

© 2026 Khanna AK et al. *JAMA Network Open*.

eFigure 6 : Percentage of patients with time beyond a MAP <75 mmHg

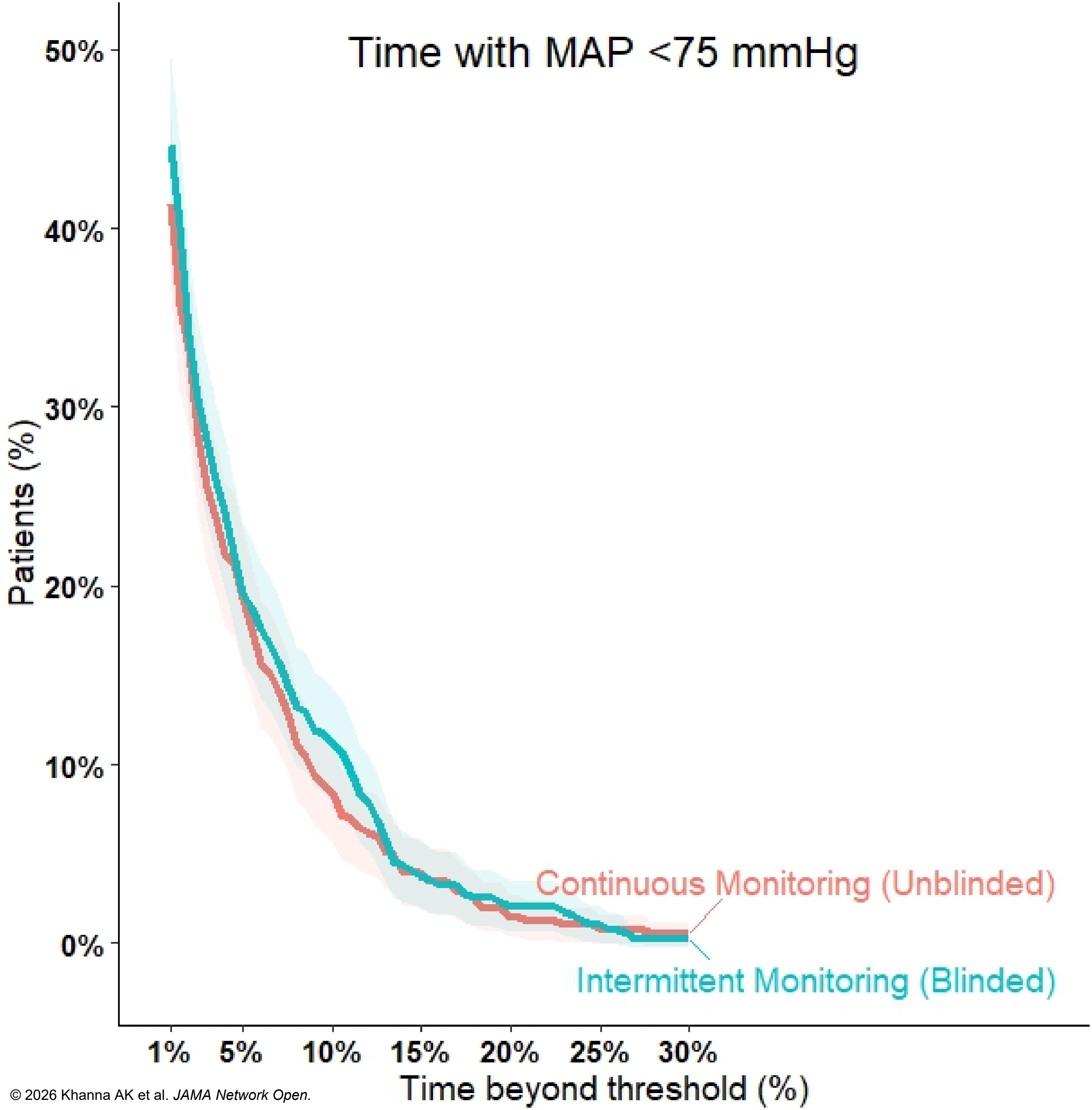

© 2026 Khanna AK et al. *JAMA Network Open.*

eFigure 7 : Percentage of patients with time beyond a MAP <80 mmHg

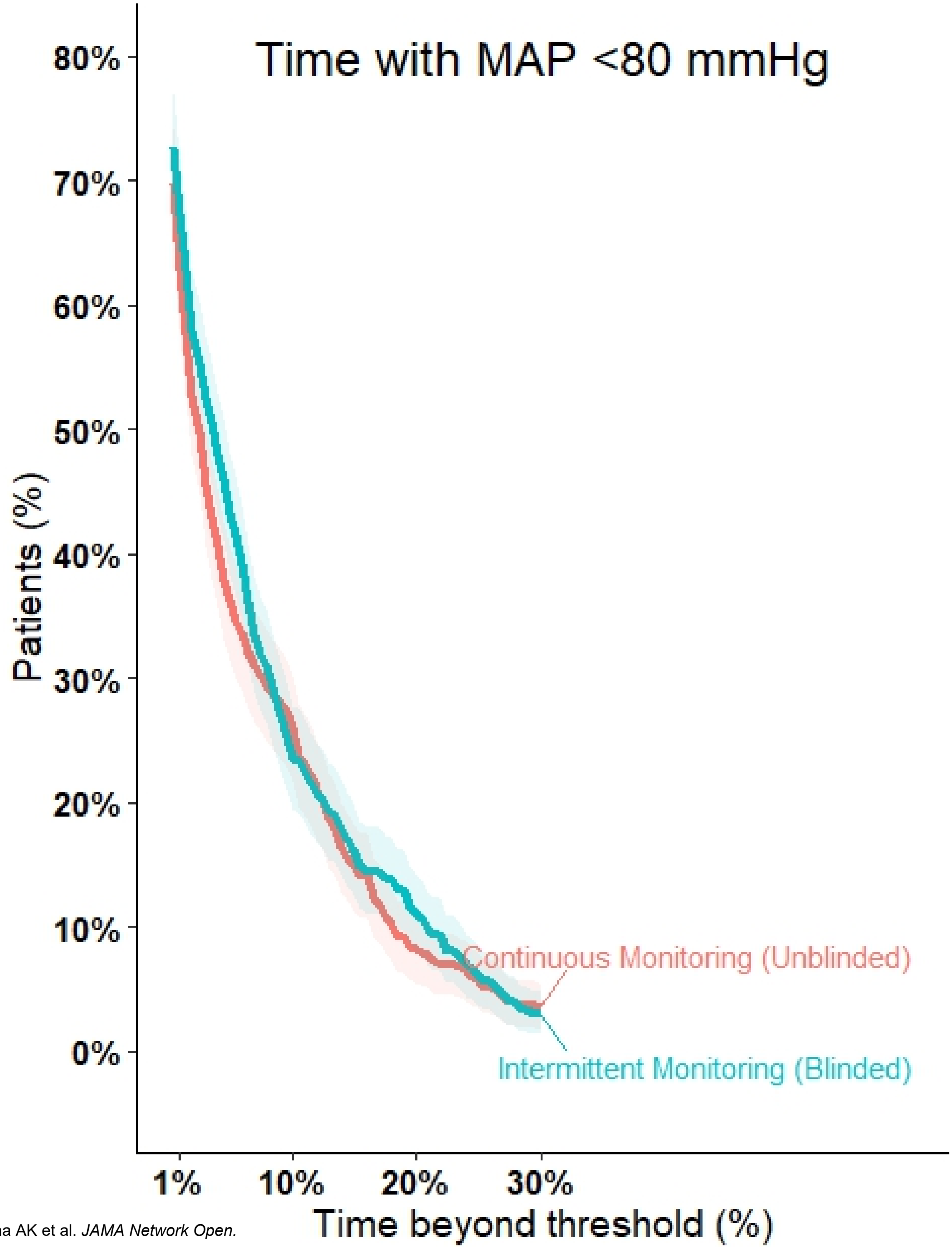

© 2026 Khanna AK et al. *JAMA Network Open.*

eFigure 8 : Percentage of patients with time beyond a MAP >130 mmHg

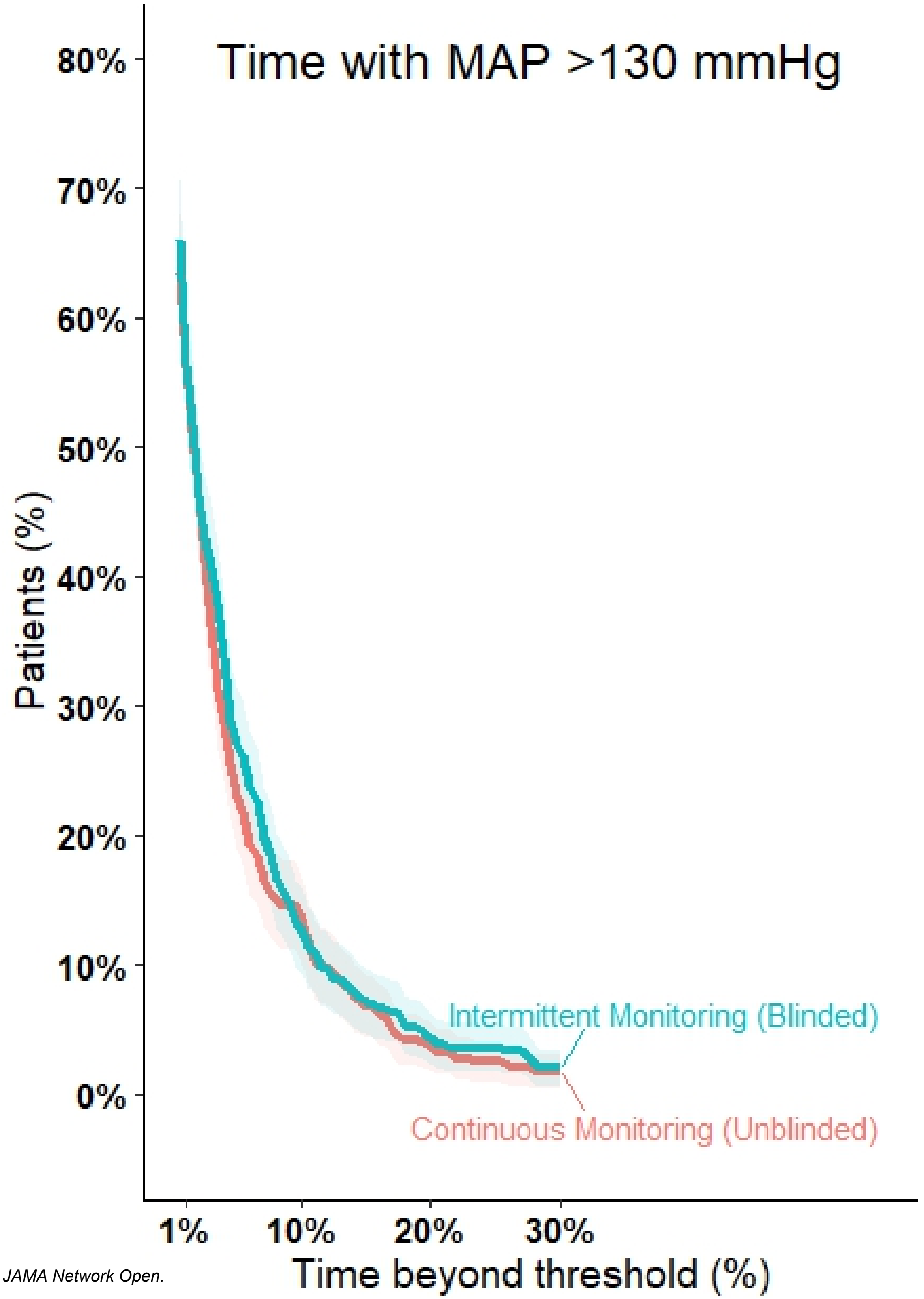

© 2026 Khanna AK et al. *JAMA Network Open.*

eFigure 9: Adjusted Relative Risk Ratios of Intervention in Continuous Monitoring Relative to Intermittent Monitoring

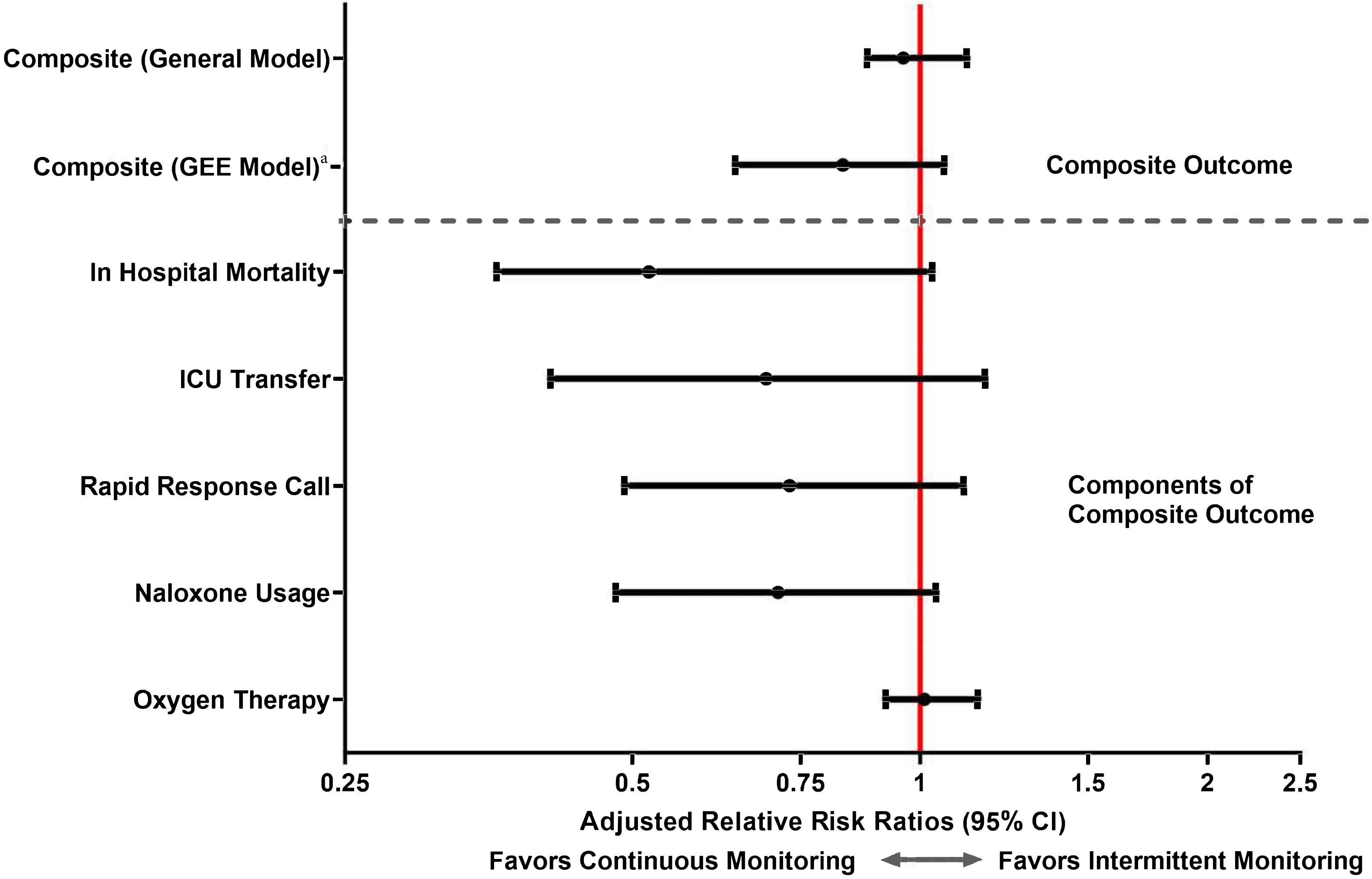

[a]Generalized linear model results adjusted for age, sex, race, diabetes, hypertension, Do-not-resuscitate order

© 2026 Khanna AK et al. *JAMA Network Open*.

eTable 1: Schematic for planned alternating interventions trial

	Weeks 1-4	Weeks 5-8	Weeks 9-12	Weeks 13-16	Weeks 17-20	Weeks 21-24	Weeks 25-28	Weeks 29-32	Weeks 33-36	Weeks 37-40	Weeks 41-44	Weeks 45-48	Weeks 49-52
Ward 1	U	B	U	B	U	B	U	B	U	U	B	U	B
Ward 2	B	U	B	U	B	U	B	U	B	B	U	B	U

U: Continuous monitoring group (unblinded)
B: Intermittent monitoring group (blinded)

eTable 2: Actual days and patients in each cluster of the alternating interventions

Periods	Days	Dates	Blinded location	Unblinded location	Blinded (n)	Unblinded (n)
1	28	10/7/2020	Ward 1	Ward 2	160	175
2	28	11/4/2020	Ward 2	Ward 1	178	150
3	36	12/2/2020	Ward 1	Ward 2	168	178
4	26	1/7/2021	Ward 2	Ward 1	150	118
5	29	2/2/2021	Ward 1	Ward 2	161	156
6	35	3/3/2021	Ward 2	Ward 1	162	194
7	28	4/7/2021	Ward 1	Ward 2	160	164
8	28	5/5/2021	Ward 2	Ward 1	161	133
9	36	6/2/2021	Ward 1	Ward 2	183	152
10	28	7/8/2021	Ward 2	Ward 1	168	159
11	27	8/5/2021	Ward 1	Ward 2	125	130
12	37	9/1/2021	Ward 2	Ward 1	169	137
Total	365	10/7/2021			1945	1846

© 2026 Khanna AK et al. *JAMA Network Open.*

eTable 3 Troponin and Myocardial Injury after Non-Cardiac Surgery (MINS)

Number of Total Days with Troponin Recorded n (%)	Continuous Monitoring (Unblinded) (N=404)	Intermittent Monitoring (Blinded Continuous) (N=394)
1	167(41.3%)	174(44.2%)
2	144(35.6%)	127(32.2%)
3	93(23%)	93(23.6%)
**Troponin Recorded per Postoperative Day, n (%)**		
1	346(85.6%)	339(86%)
2	241(59.7%)	224(56.9%)
3	153(37.9%)	144(36.5%)
**Troponin (pg/ml) Day 1**		
Median [Min, Max]	9 [2, 630]	8 [2, 2791]
**Troponin (pg/ml) Day 2**		
Median [Min, Max]	10 [2, 4331]	9 [2, 16104]
**Troponin (pg/ml) Day 3**		
Median [Min, Max]	12 [2, 2351]	12 [2, 1174]
**Average Troponin (pg/ml)**		
Median [Q1, Q3]	10 [5, 20]	10 [6, 24]
**Average Maximum daily Troponin (pg/ml)**		
Median [Q1, Q3]	11 [6, 23]	10 [6, 24]
**Myocardial Injury after Non-Cardiac Surgery (MINS) (hs-CTnI >40 pg/ml)**		
No	344(85.1%)	329(83.5%)
Yes**	60(14.9%)	65(16.5%)

**Risk ratio 1.06, 95% CI [0.65-1.24], p 0.52

© 2026 Khanna AK et al. *JAMA Network Open.*

eTable 4: Frequency of unfiltered alarms during the trial period for the set thresholds

Time Period	Vital Sign	Unfiltered Alarms/patient/24 hrs.
1st 24 Hours	SpO2 <90%	253
1st 24 Hours	SpO2 <90% in combination with other parameter alarms	258
1st 24 Hours	HR >110/minute	340
1st 24 Hours	HR >110/minute in combination with other parameter alarms	342
1st 24 Hours	MAP <65mmHg	47
1st 24 Hours	MAP <65mmHg in combination with other parameter alarms	52
24-48 Hours	SpO2 <90%	192
24-48 Hours	SpO2 <90% in combination with other parameter alarms	198
24-48 Hours	HR >110/minute	260
24-48 Hours	HR >110/minute in combination with other parameter alarms	264
24-48 Hours	MAP <65mmHg	36
24-48 Hours	MAP <65mmHg in combination with other parameter alarms	36

© 2026 Khanna AK et al. *JAMA Network Open.*
