# Supplementary material for: Continuous vs Intermittent Postoperative Vital Sign Monitoring: A Cluster Randomized Crossover Trial
Source: JAMA Netw Open. 2026 Mar 26;9(3):e263290. doi: 10.1001/jamanetworkopen.2026.3290 (PMC13022735; doi:10.1001/jamanetworkopen.2026.3290)
Supplement: Supplement 3. — Data Sharing Statement [file jamanetwopen-e263290-s003.pdf]

Data Sharing Statement

Khanna. Continuous vs Intermittent Postoperative Vital Sign Monitoring. *JAMA Netw Open*. Published March 26, 2026. doi:10.1001/jamanetworkopen.2026.3290

Data
Additional Information: https://clinicaltrials.gov/study/NCT04574908 NCT04574908
Data available: Yes
Data types: Deidentified participant data
How to access data: Ashish.Khanna@wfusm.edu
When available: With publication

Supporting Documents
Document types: Statistical/analytic code
How to access documents: Ashish.Khanna@wfusm.edu
When available: With publication

Additional Information
Who can access the data: Researchers whose proposed use of the data has been approved.
Types of analyses: Specified purpose
Mechanisms of data availability: Signed data access agreement
Any additional restrictions: Deidentified individual participant data that underlie the results reported in this article (text, tables, figures, and appendices) will be made available upon reasonable request to the corresponding author, Dr. Ashish K. Khanna (ashish.khanna@wfusm.edu), following publication. Data will be shared only in fully de-identified form and after completion of a formal data sharing agreement with Atrium Health Wake Forest Baptist / Wake Forest University School of Medicine to ensure compliance with institutional policies, legal requirements, and data privacy regulations. No patient-identifiable information will be shared, and all requests will be reviewed for ethical and regulatory compliance before access is granted.
